# Geometric and dosimetric evaluation of deep learning based auto‐segmentation for clinical target volume on breast cancer

**DOI:** 10.1002/acm2.13951

**Published:** 2023-03-15

**Authors:** Yang Zhong, Ying Guo, Yingtao Fang, Zhiqiang Wu, Jiazhou Wang, Weigang Hu

**Affiliations:** ^1^ Department of Radiation Oncology Fudan University Shanghai Cancer Center Shanghai China; ^2^ Department of Oncology, Shanghai Medical College Fudan University Shanghai China; ^3^ Shanghai Clinical Research Center for Radiation Oncology Shanghai China; ^4^ Shanghai Key Laboratory of Radiation Oncology Shanghai China

**Keywords:** auto‐segmentation, dosimetric impact, geometric metrics, radiotherapy target

## Abstract

**Background:**

Recently, target auto‐segmentation techniques based on deep learning (DL) have shown promising results. However, inaccurate target delineation will directly affect the treatment planning dose distribution and the effect of subsequent radiotherapy work. Evaluation based on geometric metrics alone may not be sufficient for target delineation accuracy assessment. The purpose of this paper is to validate the performance of automatic segmentation with dosimetric metrics and try to construct new evaluation geometric metrics to comprehensively understand the dose‐response relationship from the perspective of clinical application.

**Materials and Methods:**

A DL‐based target segmentation model was developed by using 186 manual delineation modified radical mastectomy breast cancer cases. The resulting DL model were used to generate alternative target contours in a new set of 48 patients. The Auto‐plan was reoptimized to ensure the same optimized parameters as the reference Manual‐plan. To assess the dosimetric impact of target auto‐segmentation, not only common geometric metrics but also new spatial parameters with distance and relative volume ($R_{V}$) to target were used. Correlations were performed using Spearman's correlation between segmentation evaluation metrics and dosimetric changes.

**Results:**

Only strong (|*R* ^2^| > 0.6, *p* < 0.01) or moderate (|*R* ^2^| > 0.4, *p* < 0.01) Pearson correlation was established between the traditional geometric metric and three dosimetric evaluation indices to target (conformity index, homogeneity index, and mean dose). For organs at risk (OARs), inferior or no significant relationship was found between geometric parameters and dosimetric differences. Furthermore, we found that OARs dose distribution was affected by boundary error of target segmentation instead of distance and $R_{V}$ to target.

**Conclusions:**

Current geometric metrics could reflect a certain degree of dose effect of target variation. To find target contour variations that do lead to OARs dosimetry changes, clinically oriented metrics that more accurately reflect how segmentation quality affects dosimetry should be constructed.

## 1 INTRODUCTION

Image segmentation is a key step in the radiation therapy (RT) workflow since precise delineation of the region of interest (ROI) will improve local tumor control and reduce the incidence of side effects in the surrounding normal tissues.^1^, ^2^, ^3^ Automatic segmentation approaches based on deep learning (DL) have been proven to be time‐saving and to improve consistency among oncologists, and thus greatly shorten the turnaround time of patients.^4^, ^5^, ^6^, ^7^ Recently, a lightweight DL framework was developed by using a large‐scale dataset of 28 581 cases. Superior accuracy with an average Dice of 0.95 was achieved on 67 delineation tasks and real‐time delineation in whole‐body organs at risk (OARs) and tumors was less than 2 s.^8^ Despite the great promise of this technique, it is still necessary to evaluate its geometric accuracy before implementing it in clinical applications.^9^, ^10^, ^11^

Generally, two main categories of evaluation metrics (region‐based and boundary‐based) were used for assessment of the goodness and usefulness of automatic delineation.^12^ The commonly used region‐based metrics compare the region overlap between auto‐segmentation contours and their corresponding ground truth, such as the Dice similarity coefficient (DSC)^13^ and Jaccard index (JI).^14^ Boundary‐based metrics, including Hausdorff distance (HD)^15^ and mean distance to agreement (MDA),^16^ express the difference between the boundaries of auto‐segmentation and gold standard manual contours. The abovementioned basic metrics have been shown to be an effective way to evaluate the accuracy of contouring in many studies.^12^, ^17^, ^18^ However, one issue with the commonly used metrics is that the same metric value often reflects different clinical relevant treatment outcomes, such as dosimetry and related tumor control and toxicity.^19^, ^20^, ^21^, ^22^, ^23^ Therefore, evaluation based on geometric metrics alone may not be sufficient for ROI delineation accuracy assessment. For radiotherapy, dosimetric metrics should be considered to evaluate the quality of automatic delineation results, which might help to understand the dose‐response relationship precisely from the perspective of clinical application.

To validate the performance of automatic segmentation with dosimetric indexes, several researchers have studied the correlation between contouring variation and dose differences. In the study of Kieselmann et al.,^24^ atlas‐based segmentation approaches were investigated in head and neck tumors for OARs. A weak correlation between geometric metrics and dose differences was found with *R* ^2^ < 0.5. Rooij et al.^21^ also studied the correlation between the Sørensen‐DSC and dosimetry for all OARs in the head and neck region. No single geometric index exhibited a strong correlation (*R* ^2^ = −0.24) with dosimetric differences for DL‐based auto‐segmentation methods. The correlations between geometric indices and dosimetric endpoints were low not only on nasopharyngeal but also on rectal cancer for most auto‐segmentation OARs in the study of Guo et al.^25^

Although these studies investigated the correlation between dosimetry and variation in automated delineation, they only focused on the auto‐segmentation of OARs. Intuitively, different from OARs, dose distributions around treatment targets suffer from high dose gradients. These volumes may be more sensitive to contour variation. Hence, the dose effect of target auto‐segmentation seems to be more critical to correlate with clinical outcomes. To the best of our knowledge, only one study has explored evaluating the clinically relevant outcome of target contouring variation by Xian et al.^26^ In their research, four different types of targets were selected to investigate the correlation between geometric metrics and dosimetric evaluation indices. To introduce systematic errors, sine function transformation, translation, rotation, and scaling were performed on a C‐shaped target through Python software. Except for the sine function transformation (*R* ^2^: 0.023–0.04, *p* > 0.05), the remaining three geometric transformations were correlated with D_98_ (corresponding dose of 98% volume of the target) and D_mean_ (*R* ^2^: 0.689–0.988), 80% of which exhibited *p* < 0.001.

However, in their assessment, they merely focused on dose parameters of the target, such as D_98_, mean dose (D_mean_), maximum dose (D_max_), homogeneity index (HI), and conformity index (CI). The dosimetric deviation of target contouring variation on OARs was not examined. Moreover, the investigation of the dose‐response relationship for the targets was performed on a water phantom, and the system errors of the targets were not introduced by DL‐based approaches. These transformation methods cannot represent target delineation variation between observers in clinical practice. It is therefore very difficult to reflect the clinical effect of target automatic segmentation results.

Radiotherapy after radical mastectomy is an important treatment modality for the breast cancer patients to decrease local recurrence and improve survival rate. The shape of the irradiation target is irregular, concave, and very patient‐specific. Meanwhile, the complex geometry relationship between the PTV and OARs including the ipsilateral lung and heart. The first aim of this study is to developed a DL‐based target segmentation model training on clinical radical mastectomy breast cancer cases. To evaluate the dosimetric impact of target auto‐segmentation variation, dosimetric metrics not only for targets but also for OARs were used. The correlation between commonly used geometric metrics and dose differences was analyzed comprehensively by using a new set of breast cancer patients. Additionally, to find what other characteristics can identify the variation of target delineation to OARs, we introduced two new evaluation metrics (distance‐based metrics [$D_{M}$] and relative volume [$R_{V}$]) to assess the effect of radiotherapy target auto‐segmentation. We expect this finding may contribute to a better understanding of dose‐response between target auto‐segmentation variation and the dosimetric effect, as well as what quality of automatic delineation of target is required before clinical implementing in a safe and secure way.

## 2 MATERIALS AND METHOD

**FIGURE 1:**
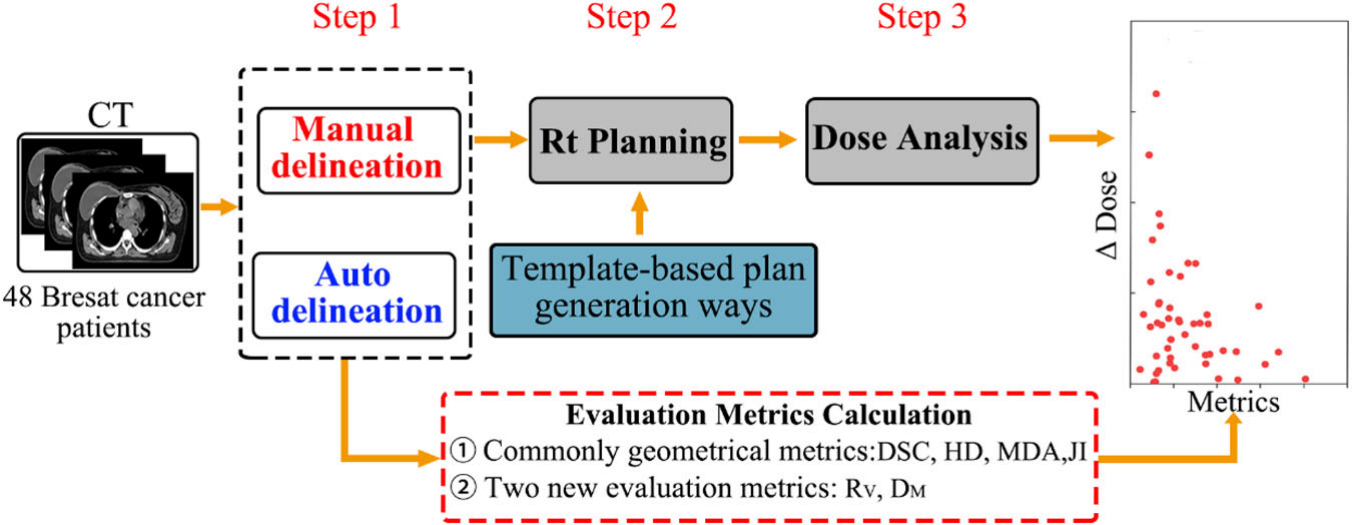
A schematic representation of this paper.

The workflow of this study is schematically depicted in Figure 1. Step 1: a DL‐based model was developed by training on 186 manual delineation data set. The resulting DL model were used to generate clinical target volume (CTV) in a new set of 48 patients. Step 2, two treatment plans were created: one based on the auto‐contours and the other based on the original manual delineation contours. Step 3, the dose difference between the reference plan and alternative plan was determined based on the dose volume histogram. To find the correlation between dosimetry and contour variation of the target, the geometric metric values were calculated for the automatically delineated planning treatment volumes (PTVs) with respect to the “gold truth.” Each of these is explained in more detail in the following section.

### 2.1 Patient data

A total of 234 modified radical mastectomy breast cancer patients were enrolled in this study. A total of 186 cases were used for developing DL‐based model and 48 for assessing the dosimetric impact of the target auto‐segmentation. All patients who had been treated with RT at Fudan University Shanghai Cancer Center between 2020 and 2021 underwent radical surgical resection of metastatic axillary lymph nodes. Simulation CT images (slice thickness 5 mm; 512 × 512 matrix) were acquired using a Philips Brilliance Big Bore multidetector‐row spiral CT scanner (Philips Healthcare, Cleveland, OH). No iodine contrast agent used for all patients. More detail of age, tumor location (left or right breast), TNM stage, and prescription of patient can be found on additional file: Supplement A. The patients were instructed to breathe freely, and no respiratory motion technologies were adopted. The treatment targets include the ipsilateral chest wall (PTV‐CW), supra/infraclavicular lymph nodes (PTV‐SCN), partial axillary lymph nodes at high risk (PTV‐ALN), and internal mammary nodes (PTV‐IMN).

### 2.2 Model architecture

**FIGURE 2:**
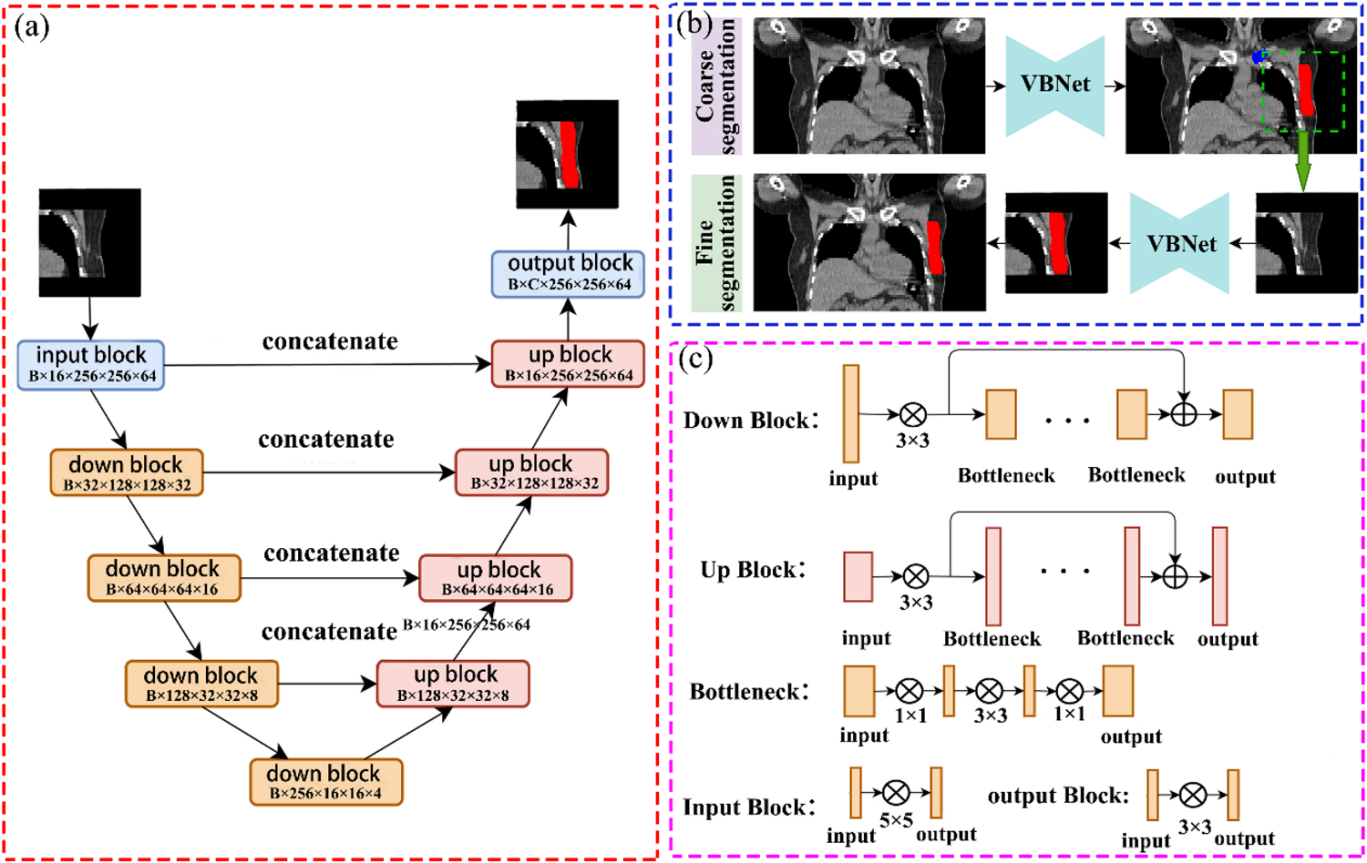
The CNN model used in our study. (a) The architecture of the VB‐based U‐net for target segmentation model. (b) The two process (coarse segmentation and fine segmentation) of this model. (c) The details of down block, up block, and bottleneck used in this U‐net.

The data set (186 cases) was partitioned into three sets (128/20/38) to obtain training, validation, and test, respectively. A cascade model was developed and is illustrated in Figure 2a–c, which could divide into coarse segmentation and fine segmentation based on VB‐Net.^27^ More detailed information about the network can be found in additional file: Supplement B.VB‐Net consists of an encoder and a decoder. In the down block of the encoder, features were first down‐sampled, then high‐level features were extracted through several bottleneck layers, and finally, the down‐sampled features and high‐level features were added for output. In the up block of the decoder, the original features were up‐sampled to recover to the original image size gradually. In the bottleneck layer, the channel of the feature will be compressed first to reduce the computation cost, and then the convolution will be used to extract high‐level features. Finally, the feature will be enlarged to the original number of channels.

The input data was CT data, and the output data were CTV and Clavicle predictions in coarse segmentation. Then, the original CT data were cropped as the input of fine segmentation to obtain the refined CTV prediction in fine segmentation. The cascade model was implemented in PyTorch, and the loss function used in the training process was the weighted average of cross‐entropy and dice loss. The Adam optimization algorithm was used to minimize the loss function, as shown in Equations (1) and (2). The Adam optimization algorithm was used to minimize the loss function, which is a variant of stochastic gradient descent optimizer.^28^

(1) $$\;loss_{cross-entropy}=-\sum_{i=1}^{C}p_{i}\log\left(q_{i}\right)\;$$

(2) $$\;loss_{dice}=-\sum_{i=1}^{C}2*\frac{p_{i}q_{i}}{p_{i}+q_{i}}\;$$

where *C* is the number of categories, $p_{i}$ is the label, and $q_{i}$ is the prediction.

### 2.3 Data augmentation and preprocessing

During coarse model training, the input CT images and labels were first down‐sampled with a ratio of [2, 2, 1], and the image and label patches of the input of coarse model with the size of [256, 256, 64] were randomly cut out from down‐sample images and labels. During fine model training, the image and label patches of the input of the fine model with the size of [256, 256, 64] were randomly cut out from the original images and labels. The following augmentation techniques were applied on the fly during coarse model and fine model training: normalization, random rotations, random scaling, and gamma correction augmentation.

In the test phase, the original CT images were first down‐sampled and normalized as the coarse model's input image to obtain the coarse predictions. The coarse predictions were interpolated to the resolution of the original images. The cropped images of the input of the fine model were cut out from the original CT images according to the boundary of coarse prediction. Specifically, the upper and lower boundaries of the cropped image are consistent with the upper and lower boundaries of the coarse prediction. The front and back and inner and outer boundaries were expanded by fifty pixels based on the boundaries of the coarse prediction, respectively. Then input the cropped image into the fine model to obtain the fine prediction.

**FIGURE 3:**
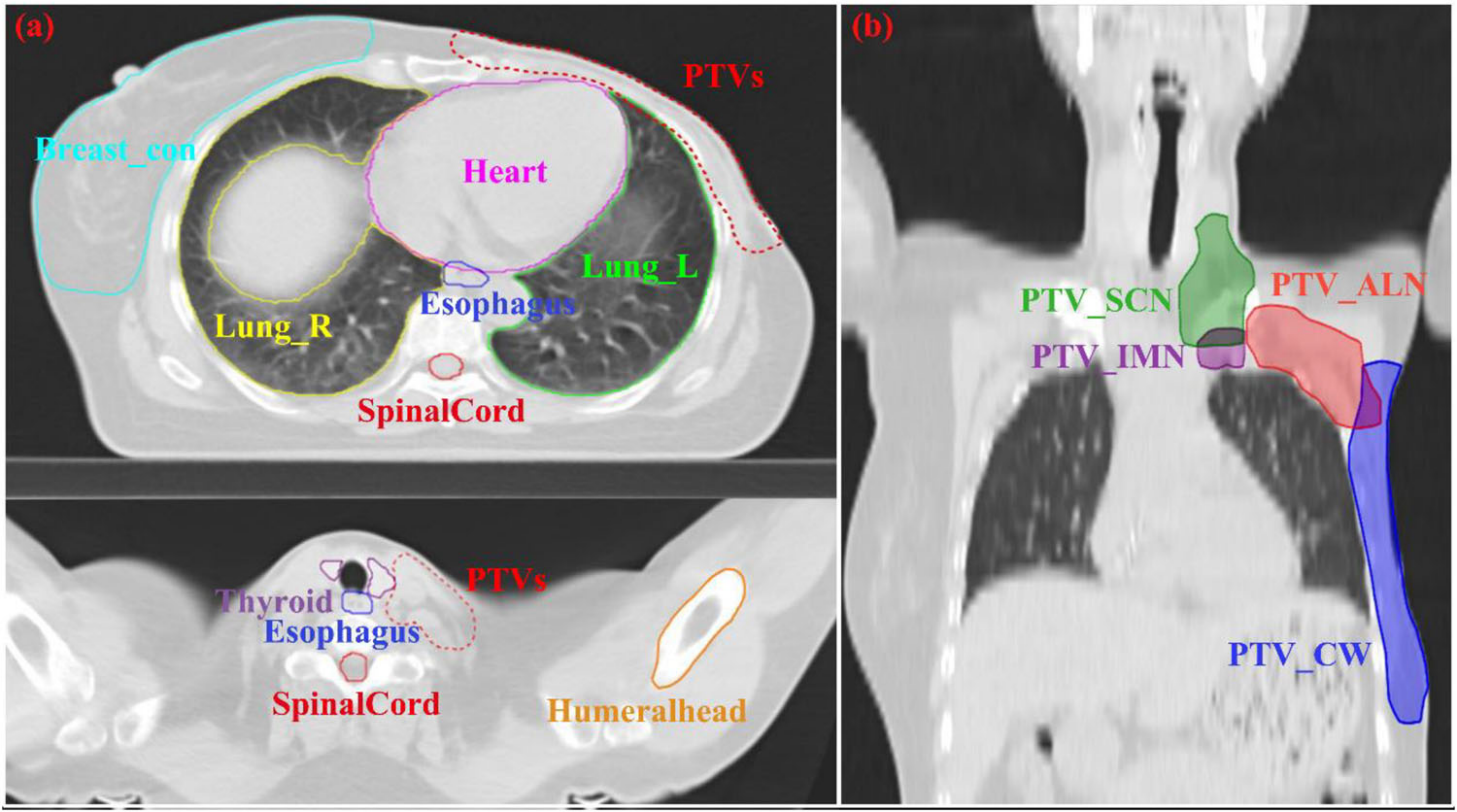
Graphical description of the OARs (a) and four separated PTVs (b) for radical mastectomy patients by oncologist.

To assess the dosimetric impact of the target auto‐segmentation method in the process of radiotherapy, we selected a new set of 48 modified radical mastectomy breast cancer cases. Manual contours, that is, manual contours were delineated by the radiation oncologist with over 5 years of experience on the United Imaging Healthcare (UIH) treatment planning system (TPS)^29^ as shown in Figure 3a,b. They contain the four targets mentioned above and OARs (contralateral breast, spinal cord, lung, esophagus, heart, thyroid, and humeral heads) presented in Table 1. The CTV segmented by a DL‐based auto‐segmentation model was marked as CTV_UIH to generate the alternative target structures, that is, auto‐contours. Considering the movement of respiratory and setup errors in the process of RT, the PTV was generated from the CTV with a uniform 5 mm margin. The two contouring sets were used as input for radiotherapy treatment plan design by using template‐based plan generation, which had the same prescription, beam setup, and optimization parameters as the reference plan. The steps of template‐based approach as follows: First, we save the manual treatment planning angles and optimization constrains as a template; Second, we load the plan template for the AI‐based contour and input these optimization parameters mentioned in our manuscript; Third, when the optimization completed, we normalize the plan (95% of the PTV received 100% of the prescription dose).

**TABLE 1:** The ROI constraint functions and dosimetric evaluation metrics.

ROI	Prescription	Constraints or objectives	Dosimetric evaluation
PTV	50 Gy/25F	*D*_95_ > Prescription, *D*_2_ < 110% Prescription, Uniform dose = Prescription	*D*_mean_, *D*_max_, *D*_min,_ CI, HI
Breast_con	/	*V*_5_ < 10%	*V*_5_
Esophagus	/	*D*_mean_ < 25 Gy	*D*_mean_
Heart	/	Mean Dose < 8 Gy	*D*_mean_
Humeralhead	/	*D*_mean_ < 25 Gy	*D*_mean_
Lung	/	*V*_5_ < 60%, *V*_20_ < 35%	*V*_5_, *V*_20_
SpinalCord	/	*D*_max_ < 45 Gy	*D*_max_
Thyroid	/	*D*_mean_ < 25 Gy	*D*_mean_

The patients were treated with a static intensity‐modulated radiotherapy (IMRT) technique with tangential fields. The optimized parameters of the clinical treatment plan are as follows: maximum number of segmented subfields (45), minimum subfield area (8 cm^2^), minimum subfield monitoring unit (8 MU), and dose calculation grid (3 mm). The prescription was 50 Gy in 25 fractions for all selected patients. After auto‐segmentation, we reoptimized the plan based on the automatically segmented PTV using a template to ensure that the same optimized parameters and functions as the reference treatment plan were set. Each patient was given two plans: Manual‐plan and Auto‐plan. The Manual‐plan was accepted and clinically approved for treatment. The Auto‐plan was reoptimized using auto‐segmented PTVs and manually delineated OARs.

### 2.4 Geometric evaluation metrics

The performance of the DL model was evaluated by commonly geometrical indices: DSC, JI, HD, and MDA. These traditional geometric metrics were calculated for the target between automatic delineation and the “gold truth.” The calculation formula of each parameter is as follows:

The DSC is parameter to measure the degree of overlap between 2 volumes (A and B). The DSC is defined as:

(3) $$\mathrm{DSC}=2*\frac{\left|A\cap B\right|}{\left|\left.A\right|\right.+\left|\left.B\right|\right.}\;$$

The DSC values range from 0, indicating no spatial overlap between the two segmentations, to 1, indicating complete overlap. The JI was calculated to obtain the overlap between contours A and B. The JI defines as:

(4) $$\mathrm{JI}=\frac{\left|A\cap B\right|}{\left|\left.A\cup B\right|\right.}$$

Values are range [0, 1], with 1 being the best value, and 0 being the worst. The HD determines the maximum distance from one point of a contour to the closest pair‐wise point of another contour. The HD is defined as:

(5) $$\mathrm{HD}\left(A,B\right)=\max\left\{H\left(A,B\right),\;H\left(B,A\right)\right\}$$

(6) $$H\left(A,B\right)={}_{a\in A}^{max}\left\{{}_{b\in B}^{min}\left\{d\left(a,b\right)\right\}\right\}$$

where *d*(*a*,*b*) represents the 3D Hausdorff distance between point a from contour A and point *b* from contour B. The MDA was used to quantify the mean 3D distances between contours A and B. The definition is as follows:

(7) $$\mathrm{MDA}\left(A,B\right)=\frac{H{\left(A,B\right)}_{mean}+H{\left(B,A\right)}_{mean}}{2}$$

(8) $$\;H{\left(A,B\right)}_{mean}={}_{a\in A}^{mean}\left\{{}_{b\in B}^{min}\left\{d\left(a,b\right)\right\}\right\}$$

For a perfect overlap between A and B, the values of MDA are 0. For an imperfect overlap, the values of MDA are large.

The same geometric metrics value of contouring may represents different dose effect.^30^ To understand the relationship between dosimetry and contour variation, several spatial parameters, including location to target, size and shape, for alternative OARs contours were introduced by Poel et al.^31^ They found that the dose effect is more susceptible to OAR contour variation with respect to the direction rather than the relative location to the target. Therefore, in this study, two new evaluation metrics ($R_{V}$ and $D_{M}$) with spatial information were designed on to examine the correlation between geometric metrics and clinical dosimetric indices to OARs. The $R_{V}$ is the volume ratio of the ROI and PTV as following equation:

(9) $$\;\;R_{V}=\frac{V_{ROI}}{V_{PTV}}$$

The value of $R_{V}$ was used to measure the $R_{V}$ between OAR and PTV. The $D_{M}$ is the mean Hausdorff distance between the OARs and PTV contours as the following formula:

(10) $$\;D_{M}=\;\mathrm{MDA}\left(\mathrm{OAR},\;\mathrm{PTV}\right)$$

The value of $D_{M}$ represents the relative distance between the OAR and PTV volumes.

To further identify the boundary error of target auto‐segmentation to OARs dose distribution, the exclusive OR (XOR) was perform on Auto‐PTV and Manual‐PTV. XOR is a mathematical operator that applies to logical operations. If the values of A and B are different, the XOR result is 1. If the values A and B are the same, the XOR result is 0. It is our hypothesis that the dose difference between Auto‐plan and Manual‐plan was due to boundary contour difference. Therefore, the XOR operator was introduced to further find what other characters have an effect on OARs dose difference for two sets of target contours with same DSC value.

### 2.5 Dosimetric evaluation indices

The dose analysis was processed between the Manual‐plan and Auto‐plan to evaluate the dosimetric differences. The dosimetric evaluation metrics for OARs are the maximum dose for serial organs and the mean dose or volume areas receiving radiation for parallel organs. For targets, the *D*_mean_, *D*_max_, *D*_min_, HI,^32^ and CI^33^ were used. All the dosimetric indices are listed in Table 1. The CI and HI were calculated using the following formulas:

(11) $$\mathrm{HI}=\frac{D_{5}}{D_{95}}\;$$

(12) $$\mathrm{CI}=\frac{V_{R}*V_{R}}{V_{T}*V_{dose}}$$

where *D_X_* is the corresponding dose of X% volume of the target. $V_{T}$, $V_{dose}$ are the volume of target and the reference isodose line, respectively, and $V_{R}$ is the volume of target covered by reference isodose line.

### 2.6 Statistical analysis

Two‐tailed (95% confidence interval) nonparametric Spearman correlation tests were performed using IBM SPSS Statistics 23.0 (IBM SPSS Inc., Chicago, IL, USA). For comparisons of reference and alternative groups, paired t‐tests and Wilcoxon signed‐rank tests were used for statistical analysis, with *p* values less than 0.05 regarded as statistically significant.

## 3 RESULTS

### 3.1 DL model performance evaluation

**FIGURE 4:**
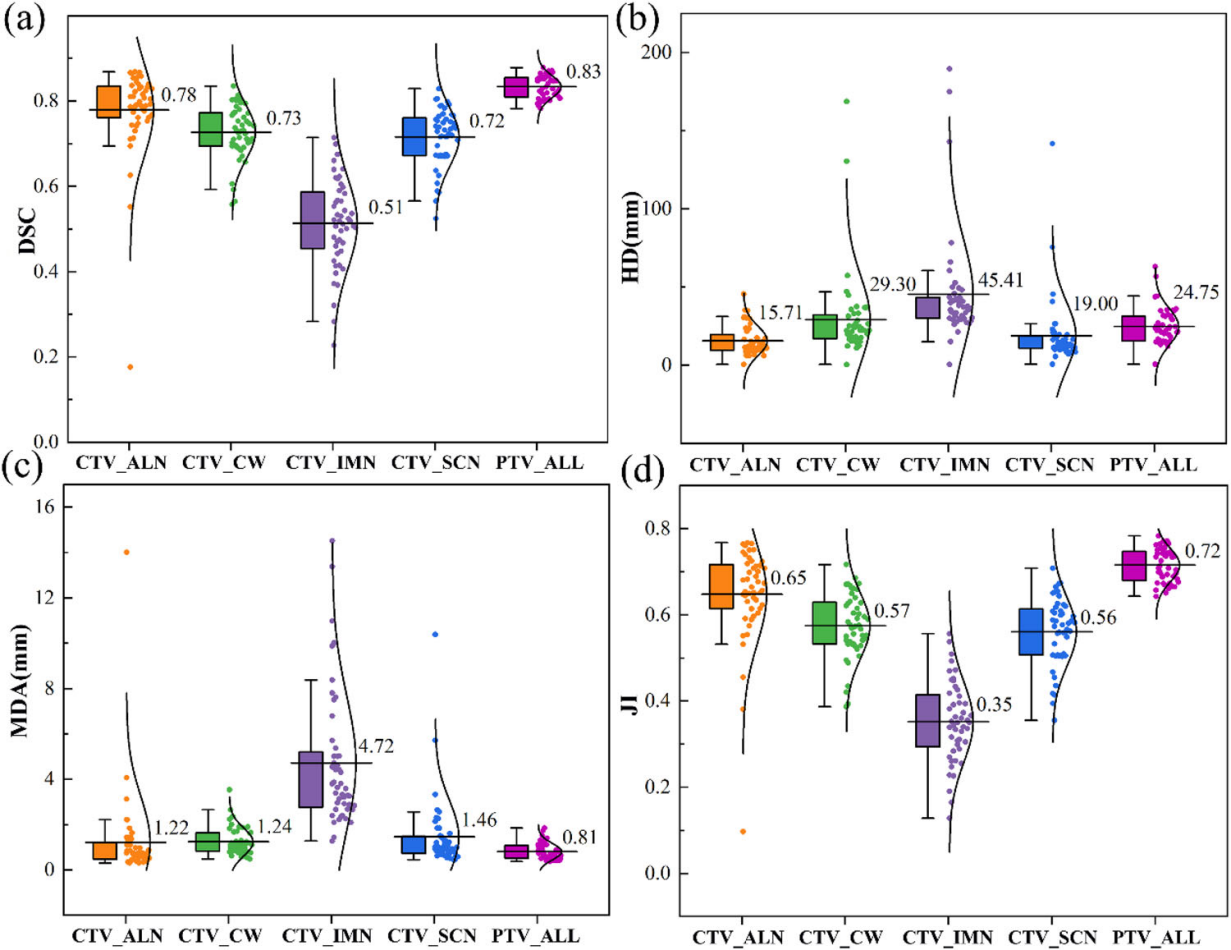
Geometric metrics results of target auto‐segmentation model for CTV_ALN (axillary lymph nodes), CTV_CW (chest wall), CTV_IMN (internal mammary nodes), CTV_SCN (supra/infraclavicular lymph nodes), and the PTV_ALL. Colored box plots display the mean values with the interquartile range, and the distribution of corresponding values show on right. (a) the DSC, (b) the HD, (c) the MDA, and the JI (d). The data can be found in additional file: Supplement C.

Figure 4 represents the box plots and values distribution of the four‐evaluation metrics (DSC, HD, MDA, and JI) for DL model. For DSC, it can be seen that the target segmentation model performed relative well (mean DSC > 0.7) in terms of CTV_ALN, CTV_CW, and CTV_SCN. Only the small volume CTV_IMN had lower values (mean DSC = 0.51). The mean DSC of PTV_ALL is up to 0.83 after adding the movement of respiratory and setup errors margin on CTV. For other three indices (HD, MAD, and JI), the same conclusion results could also be drawn at Figure 4b–d.

### 3.2 Correlation between the dose effect and traditional geometric metrics

**FIGURE 5:**
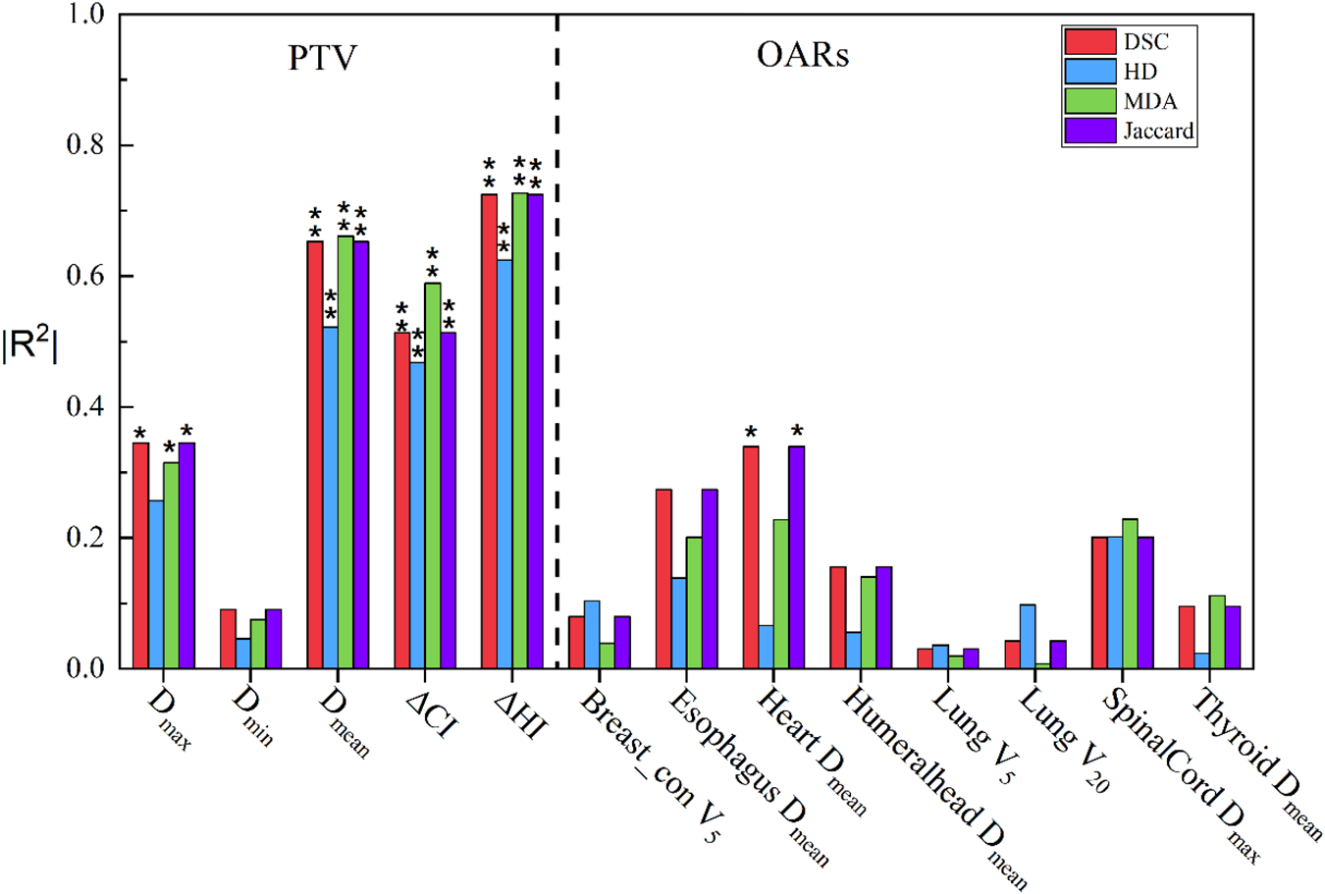
Spearman correlation between dosimetric differences versus DSC, HD, MDA, and JI (two‐tailed). **p < 0.01, *p < 0.05. The data are presented in additional file: Supplement D.

Figure 5 shows the results of the correlation analysis between dosimetric effect and traditional geometric parameters. For PTV, the strong correlations were exhibited between the ΔHI and all four geometric metrics (DSC:|*R* ^2^| = 0.725, *p* < 0.01, HD:|*R* ^2^| = 0.625, *p* < 0.01, MDA:|*R* ^2^| = 0.727, *p* < 0.01, JI:|*R* ^2^| = 0.725, *p* < 0.01). Superior correlations (|*R* ^2^| > 0.6) were found between mean dose to PTV in Manual‐plan and Auto‐plan with respect to DSC (|*R* ^2^| = 0.64, *p* < 0.01), MDA (|*R* ^2^| = 0.662, *p* < 0.01) and JI (|*R* ^2^| = 0.64, *p* < 0.01). The correlation coefficients for ΔCI to four geometric indices and mean dose to HD were greater than 0.4 but less than 0.6, showing a moderate correlation. Weak (0.2 < |*R* ^2^| < 0.4) or very weak (|*R* ^2^| < 0.2) correlations were found for *D*_max_ and *D*_min_ of PTV. For OARs, an inferior relationship or no significant was found between geometric parameters and dosimetric differences. The largest correlation coefficient was 0.287 (*p* = 0.048) for DSC and JI to heart.

### 3.3 Correlation between dosimetry of ROI and spatial parameter evaluation metrics

**FIGURE 6:**
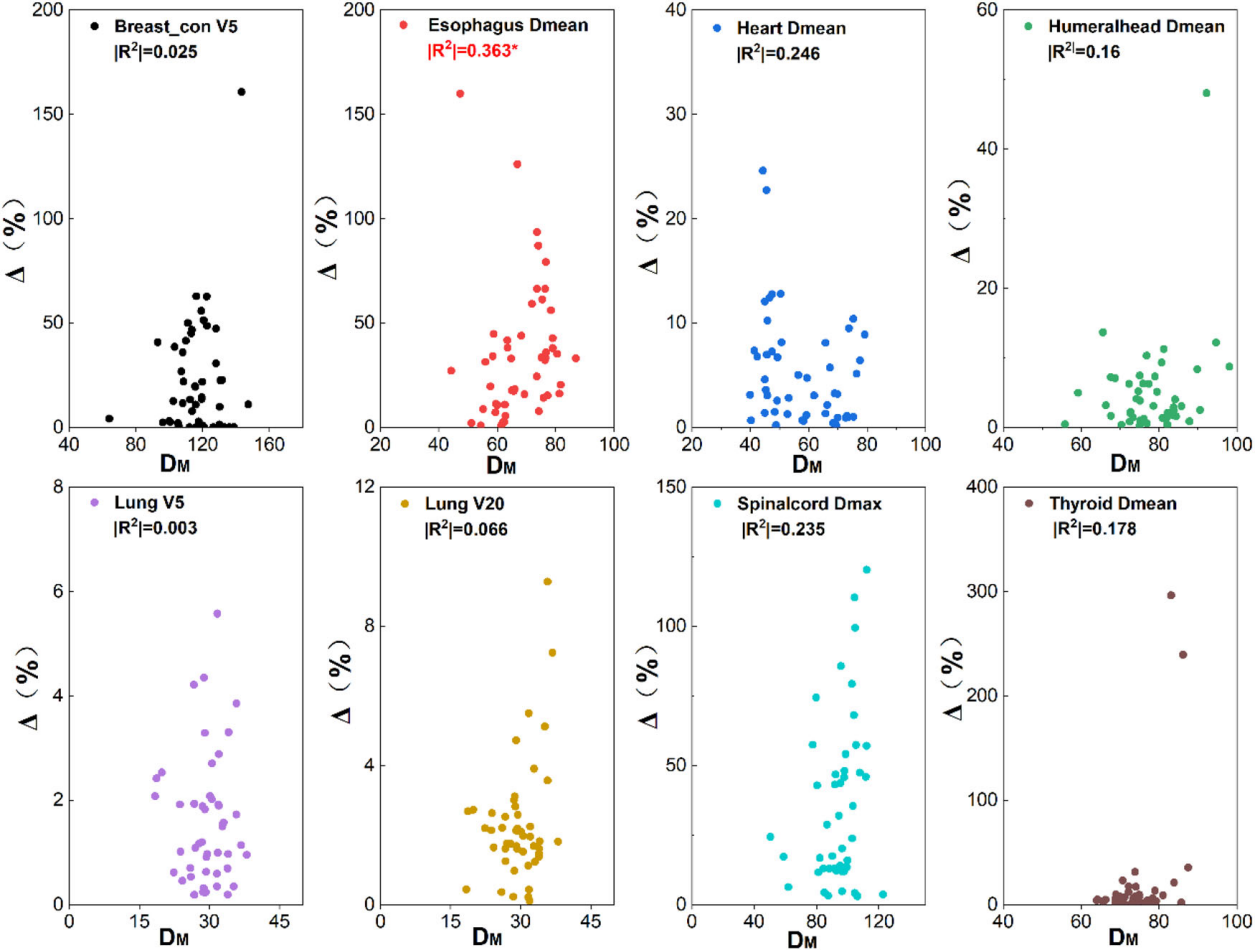
Spearman correlation between *R_V_* and dosimetric differences to OARs (two‐tailed). **p < 0.01, *p < 0.05.

**FIGURE 7:**
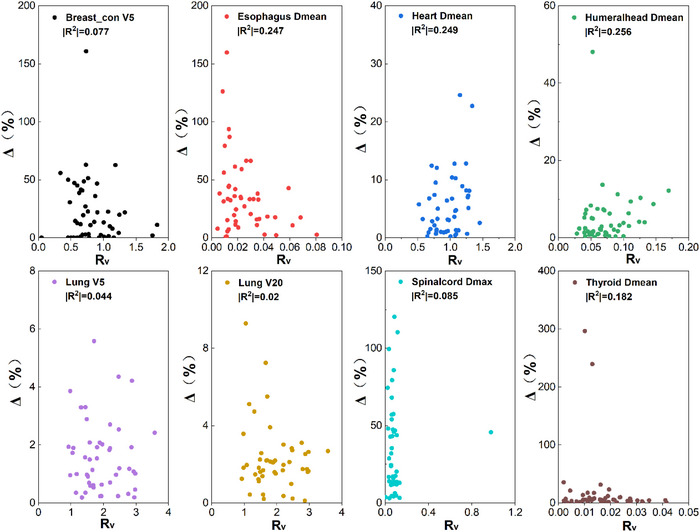
Spearman correlation between *D_M_* and dosimetric differences to OARs (two‐tailed). **p < 0.01, *p < 0.05. Data can be found in additional file: Supplement E.

To further examine the correlation between the dose differences to OAR and geometric indices. Two new evaluation metrics ($R_{V}$ and $D_{M}$) with spatial information were used in this paper. The correlation between these two geometric metrics and clinical dosimetric difference is shown in Figures 6, 7. No significant correlation was found between the two new evaluation metrics and dosimetric difference to OAR. The largest correlation coefficient was 0.363 (*p* < 0.05) between D_M_ and mean dose to Esophagus.

**FIGURE 8:**
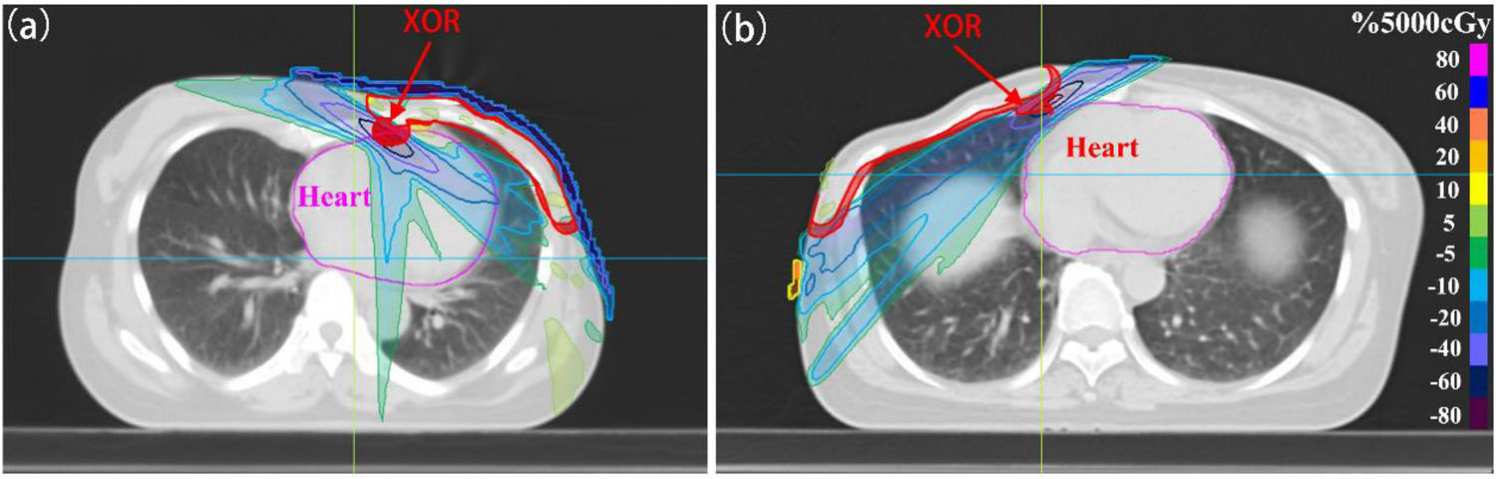
Dose‐distribution of two clinical breast cancer cases (prescription = 5000 cGy) with same DSC of PTV_ALL. The red‐shaded area represents the operation of exclusive OR for manual PTV and Auto‐PTV. (a) More overlap between heart and XOR. (b) Less overlap between heart and XOR.

To find how the impact of target auto‐segmentation to OARs dose distribution, two breast cancer patient with same DSC was used. The XOR was perform on Auto‐PTV and Manual‐PTV. The dose distributions differences of these two plans are shown in Figure 8. For heart, it is noted that there was considerable variation in the dose distribution of heart for the same DSC. The more overlap between heart and XOR, the higher the mean dose change of heart. This means that the same metric value (DSC) often represents different dose distributions depending on the location of XOR. DSC may not show a strong correlation with the dosimetric variation of OARs. The location of variation of target segmentation play a decisive role for dosimetric difference for OARs.

## 4 DISCUSSION

It is commonly presumed that quantification of the degree of variation or uncertainty of the contouring through geometric metrics is important, while several studies have indicated that determining the clinical impact through dosimetric indices remains important. These studies have investigated the dosimetric impact of auto‐segmentation to OARs and tried to clarify the correlation between auto‐segmentation geometric indices and dosimetric effects. However, most researchers found that contour variation has no significant impact on the corresponding dose evaluation metrics, and the relationship between the geometric metrics and dosimetric endpoints was non‐monotonic for most OARs.^21^, ^24^, ^25^ Dosimetrically, the advanced technologies of radiation treatment, such as IMRT and Volumetric Modulated Arc Therapy (VMAT), allow steeper dose gradients around the target margin to decrease the irradiated volume of OARs, and therefore, these areas are much more susceptible to contour variation. This may be the reason why no or a weak correlation can be found between dosimetry and OARs construing variation. In view of this, the relationship between geometric metrics and dosimetric endpoints for targets is still the focus of the current study and needs to be investigated in more depth.

To assess the dosimetric impact of target auto‐segmentation, systematic and random errors were introduced for contouring through Python software by Xian et al.^26^ They found that translation, scaling, and rotation transformation were superiorly correlated with the dose differences, but for sine function transformation, the correlations were inconsistent. This indicates that the method of transformation for the target is important and can also significantly affect the clinical metric values. Moreover, these transformation methods were not introduced by DL‐based methods and cannot simulate the inter‐ and intraobserver differences for oncologists. The other limitation of their study is that they only focused on PTV evaluation metrics. The effect of target contouring variation on OARs dosimetric differences was not investigated. In this study, we investigated the dosimetric effects of auto‐segmentation not only for targets but also for OARs using clinical breast cancer cases. We found that the commonly used geometric metrics only have strong relationship with CI, HI and mean dose to PTV, as shown in Figure 5. This is easy to understand because these three evaluation indices were more susceptible to contouring change of target. CI reflects the geometrical characterization of PTV and contouring variation have a major impact on dose distribution of target which than can induce change of HI and mean dose. For other dosimetric metrics (D_max_ and D_min_), these two values are determined by optimization parameters of treatment plan design, which changed very little between alternative and reference contours. Similar conclusions were obtained in the research of Xian et al.^26^

**FIGURE 9:**
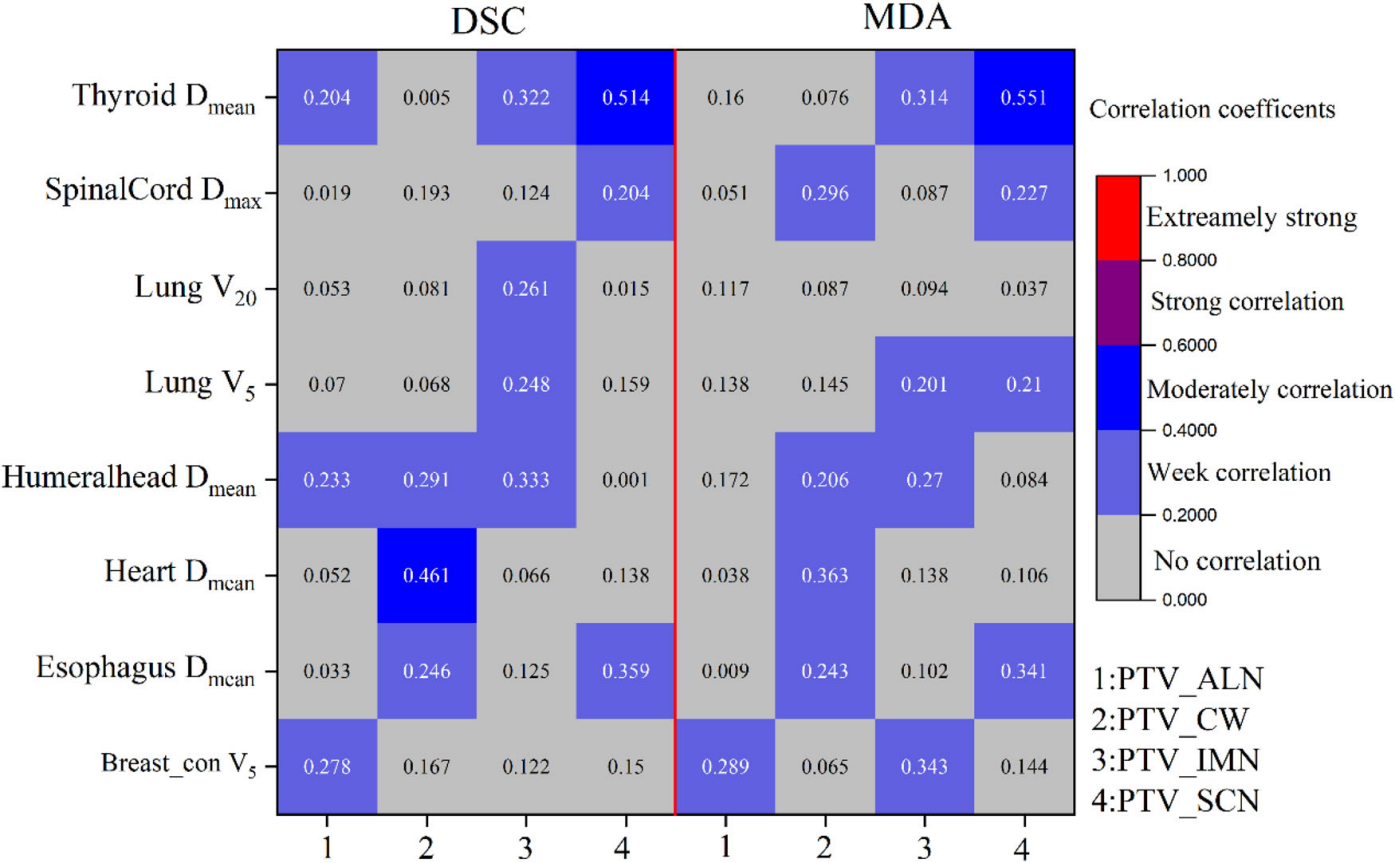
Pearson correlation coefficients between dosimetric difference and two evaluation metrics (DSC and MDA) for four separated PTV. *p < 0.05, **p < 0.01.

For OARs, the strong correlation between the traditional evaluation metrics and dose difference was not found as shown in Figure 5. We also investigated the correlation between the four separated PTVs and dosimetric difference to OARs. The results are graphically represented in Figure 9. It can be seen from the results that a moderately correlation can be found between mean dose to heart and DCS for PTV_CW. For DSC and MDA, the moderately correlation was established between mean dose to thyroid and PTV_SCN. However, the strong relationship between the dosimetric difference and OARs evaluation metrics remains unrevealed only used four traditional geometric metrics.

From the study of Robert Poel et al.,^31^ not only several geometrically based metrics, such as DSC and HD, but also other spatially related metrics (size, shape, and relative location to the target) were included. They found that (1) the organ‐based analysis revealed that there was a better correlation for the larger OARs than for the smaller OARs and (2) the direction of the contour variation with respect to the relative location of the target indices had a greater correlation with the dose effect. Based on these two points, we attempt to construct two new evaluation metrics: $D_{M}$ and $R_{V}$ to find the high‐level relationships between dosimetry and evaluation metrics.

From Figures 6, 7, it is noted that no significant correlation was found between these two new metrics and dose differences for most OARs. The *D_M_* and the size of OAR to target are not the key factor to influence the dose effect for OAR. To further investigate the correlation between target auto‐segmentation variation and evaluation metrics, the exclusive OR was performed on Auto‐PTV and Manual‐PTV. The dose distributions difference of these two plans with same DSC are shown on Figure 8. We know that the more overlap between heart and XOR, the higher dose change to OAR. It means that the distance from a target dose not directly influence the dose effect. The reason that can account for this is that the location information of target variation was not clarified, even though the volume and distance information were introduced to the two new traditional geometric metrics. For OAR, the boundary error of target segmentation plays a decisive role to dose difference of ORAs, namely, the location of target variation is the key factor.

In this study, we attempt to find metrics that reflect dosimetry and the degree of acceptability for auto‐segmentation of the target. The new spatial parameter metrics introduced geometric characteristics and more spatial parameters, such as the relative distance and volume to the target. The present study still has several limitations that need to be addressed in future investigations. First, this new metric is only used for one type of cancer. We should use it to perform more analysis for multi‐prescription nasopharyngeal cancer because of the complex form of size, shape and location of the target and OARs. Second, in this paper, only two new valuation metrics were used to find a strong relationship between dosimetry and contouring variation.

## 5 CONCLUSION

In conclusion, we successfully developed a VB‐Net target segmentation model and the dosimetric effect of auto‐delineation variation of PTV was analyzed on clinical radical mastectomy breast cancer cases. Our results demonstrated that the common geometric metrics are well correlated with dosimetric assessment parameters CI, HI, and mean dose to PTV. For OARs, the correlation between dose differences and the geometric metrics to OARs was weak. To find target contour variations that do lead to OARs dosimetry changes, other spatial parameters, such as distance based and $R_{V}$ to target metrics, were introduced to construct new assessment indices. We found that dose distribution of OARs was affected by boundary error of target segmentation instead of distance and $R_{V}$ to target. These results suggest that the current commonly used geometric evaluation metric for target segmentation could reflect a certain degree of geometric similarity. To accurately reflect how segmentation quality affects dosimetry more clinically oriented metrics should be constructed in future research.

## AUTHOR CONTRIBUTIONS

*Study concept and design*: Yang Zhong, Ying Guo, Jiazhou Wang, and Weigang Hu. *Acquisition of data*: Yang Zhong and Ying Guo. *Analysis and interpretation of data*: Yang Zhong, Ying Guo, and Yingtao Fang. *Statistical analysis*: Yang Zhong, Ying Guo, and Zhiqiang Wu. *Drafting of the manuscript*: Yang Zhong, Ying Guo, Jiazhou Wang, and Weigang Hu. All authors read and approved the final manuscript.

## CONFLICT OF INTEREST STATEMENT

The authors declare that they have no competing interests.

## ETHICS STATEMENT

This study was approved by the Fudan University Shanghai Cancer Center Institutional Review Board and all methods were performed in accordance with the guidelines and regulations of this ethics board. Informed consent was obtained from all individual participants included in the study.

## Supporting information



Supporting Information

Fig. 2 The CNN model used in our study. (a) The architecture of the VB‐based U‐net for target segmentation model. (b)The two process (coarse segmentation and fine segmentation) of this model. (c) The details of down block, up block, and bottleneck used in this U‐net.

Supporting Information

Supporting Information

Supporting Information

## Data Availability

The datasets used and/or analyzed during the current study are available from the corresponding author on reasonable request.
