# Supplementary material for: Geometric and dosimetric evaluation of deep learning based auto‐segmentation for clinical target volume on breast cancer
Source: J Appl Clin Med Phys. 2023 Mar 15;24(7):e13951. doi: 10.1002/acm2.13951 (PMC10338811; doi:10.1002/acm2.13951)
Supplement: Supplementary file 1 — Supporting Information [file ACM2-24-e13951-s005.docx]

| **RT Number** | **Age** | **Diagnosis** | **TNM stage** | **Radiotherapy (C=PTV-CW, S=PTV-SCN,I= PTV-IMN,A=PTV-ALN)** | **Prescription** | **Radiation Technology** |
| --- | --- | --- | --- | --- | --- | --- |
| RT014017 | 45 | MRM | T1N3M0 | Right：C+S+I | 50Gy/25Fx | IMRT |
| RT014099 | 69 | MRM | T2N2M0 | Left：C+S+I | 50Gy/25Fx | IMRT |
| RT014263 | 56 | MRM | T1N3M0 | Left：C+S+I | 50Gy/25Fx | IMRT |
| RT014338 | 42 | MRM | T2N1M0 | Left：C+S+I | 50Gy/25Fx | IMRT |
| RT014432 | 47 | MRM | ypT0N2M0 | Right：C+S+I | 50Gy/25Fx | IMRT |
| RT014482 | 54 | MRM | T2N1M0 | Left：C+S+I | 50Gy/25Fx | IMRT |
| RT014765 | 60 | MRM | T2N1M0 | Left：C+S+I | 50Gy/25Fx | IMRT |
| RT014753 | 39 | MRM | T2N1M0 | Right：C+S+I | 50Gy/25Fx | IMRT |
| RT014819 | 37 | MRM | ypT2N1M0 | Right：C+S+I | 50Gy/25Fx | IMRT |
| RT014990 | 35 | MRM | T2N2M0 | Right：C+S+I | 50Gy/25Fx | IMRT |
| RT015074 | 49 | MRM | T2N1M0 | Right：C+S+I | 50Gy/25Fx | IMRT |
| RT014917 | 59 | MRM | T2N3M0 | Right：C+S+I | 50Gy/25Fx | IMRT |
| RT015121 | 60 | MRM | ypT2N1M0 | Left：C+S+I | 50Gy/25Fx | IMRT |
| RT015125 | 27 | MRM | T3N0M0 | Right：C+S+I | 50Gy/25Fx | IMRT |
| RT015387 | 46 | MRM | T2N3M0 | Right：C+S+I | 50Gy/25Fx | IMRT |
| RT015389 | 47 | MRM | ypT0N0M0 | Right：C+S+I | 50Gy/25Fx | IMRT |
| RT015442 | 41 | MRM | T1N3M0 | Right：C+S+I | 50Gy/25Fx | IMRT |
| RT015495 | 50 | MRM | T3N3M0/ypTxN3M0 | Left：C+S+I | 50Gy/25Fx | IMRT |
| RT015573 | 67 | MRM | T2N1M0 | Right：C+S+I | 50Gy/25Fx | IMRT |
| RT015385 | 50 | MRM | ypT1N2M0 | Left：C+S+I | 50Gy/25Fx | IMRT |
| RT015833 | 41 | MRM | T3N0M0 | Left：C+S+I | 50Gy/25Fx | IMRT |
| RT015843 | 48 | MRM | T1N1M0 | Right：C+S+I | 50Gy/25Fx， | IMRT |
| RT016141 | 62 | MRM | T2N1M0 | Left：C+S+I | 50Gy/25Fx | IMRT |
| RT016140 | 44 | MRM | T2N1M0 | Left：C+S+I | 50Gy/25Fx | IMRT |
| RT016160 | 58 | MRM | T2N3M0/ypT0N0M0 | Left：C+S+I | 50Gy/25Fx | IMRT |
| RT016128 | 55 | MRM | T2N1M0 | Left：C+S+I | 50Gy/25Fx | IMRT |
| RT016336 | 45 | MRM | ypT3N2M0 | Left：C+S+I | 50Gy/25Fx+10Gy/5Fx | IMRT |
| RT016326 | 61 | MRM | T3N2M0/ypT2N1M0 | Right：C+S+I+A | 50Gy/25Fx+10Gy/5Fx | IMRT |
| RT016324 | 52 | MRM | T2N1M0/T2N0M0 | Left：C+S+I | 50Gy/25Fx | IMRT |
| RT016334 | 63 | MRM | T1N1M0 | Left：C+S+I | 50Gy/25Fx | IMRT |
| RT016478 | 43 | MRM | T2N1M0 | Right：C+S+I | 50Gy/25Fx | IMRT |
| RT016541 | 47 | MRM | T2N1M0/ypT0N0M0 | Right：C+S+I | 50Gy/25Fx | IMRT |
| RT016562 | 66 | MRM | T2N3M0 | Right：C+S+I+A | 50Gy/25Fx+10Gy/5Fx | IMRT |
| RT016557 | 67 | MRM | ypT2N2M0 | Left：C+S+I+A | 50Gy/25Fx | IMRT |
| RT016705 | 53 | MRM | T1N3M0 | Left：C+S+I+A | 50Gy/25Fx | IMRT |
| RT016223 | 57 | MRM | T1N1M0 | Right：C+S+I+A | 50Gy/25Fx | IMRT |
| RT016804 | 56 | MRM | cT2N2M0 | Left：C+S+I+A | 50Gy/25Fx+IMNboost 10Gy/5Fx | IMRT |
| RT016814 | 55 | MRM | cT2N3M0 | Left：C+S+I+A | 50Gy/25Fx | IMRT |
| RT016746 | 32 | MRM | cT2cN2cM0 | Right：C+S+I+A | 50Gy/25Fx | IMRT |
| RT017301 | 35 | MRM | T2N2M0 | Right：C+S+I+A | 50Gy/25Fx | IMRT |
| RT017299 | 64 | MRM | T2N1M0 | Left：C+S+I+A | 50Gy/25Fx | IMRT |
| RT017292 | 53 | MRM | T2N3M0 | Right：C+S+I+A | 50Gy/25Fx+10GY/5Fx | IMRT |
| RT017291 | 56 | MRM | T2N2M0 | Right：C+S+I+A | 50Gy/25Fx+IMNboost 10Gy/5Fx | IMRT |
| RT017494 | 61 | MRM | ypT0N2M0 | Right：C+S+I | 50Gy/25Fx | IMRT |
| RT019636 | 67 | MRM | pT2N1M0 IIB | Left：C+S+I | 50Gy/25Fx | IMRT |
| RT019744 | 67 | MRM | pT2N1M0 IIB | Right：C+S+I | 42.6Gy/16Fx | IMRT |
| RT019990 | 39 | MRM | T1N1M0 | Right：C+S+I | 50Gy/25Fx | IMRT |
| RT019998 | 52 | MRM | T1N1M0 | Right：C+S+I | 50Gy/25Fx | IMRT |
| RT019894 | 64 | MRM | ypT0N0M0 | Left：C+S+I | 50Gy/25Fx | IMRT |
| RT020225 | 46 | MRM | pT1N0M0 | Left：C+S+I | 50Gy/25Fx+0Gy/5Fx | IMRT |
| RT020427 | 61 | MRM | pT1N1M0 IIA | Left：C+S+I | 50Gy/25Fx | IMRT |
| RT020428 | 70 | MRM | pT2N1M0 IIB | Left：C+S+I | 42.6Gy/16Fx | IMRT |
| RT020883 | 31 | MRM | T2N1M0 | Right: C+S+I | 50Gy/25Fx | IMRT |
| RT020441 | 36 | MRM | cT2N1M0/ypT1aN0M0 | Left：C+S+I | 50Gy/25Fx | IMRT |
| RT021103 | 54 | MRM | pT2N3M0 | Left：C+S+I | 50Gy/25Fx+14Gy/7Fx | IMRT |
| RT021158 | 48 | MRM | pT3M2M0 | Left：C+S+I | 50Gy/25Fx | IMRT |
| RT021336 | 31 | MRM | pT2N2M0 IIIA | Right：C+S+I | 42.6Gy/16Fx | IMRT |
| RT021332 | 40 | MRM | T2N1M0 | Left：C+S+I | 50Gy/25Fx | IMRT |
| RT021518 | 58 | MRM | T2N1M0 | Right：C+S+I | 50Gy/25Fx | IMRT |
| RT021495 | 61 | MRM | T1bN2M0 | Left：C+S+I | 50Gy/25Fx | IMRT |
| RT020817 | 39 | MRM | pT3N3M0 | Right：C+S+I | 50Gy/25Fx+10Gy/5Fx | IMRT |
| RT021734 | 51 | MRM | T2N1M0 | Left：C+S+I | 42.6Gy/16Fx | IMRT |
| RT021947 | 50 | MRM | T2N1M1 | Left：C+S+I | 50Gy/25Fx | IMRT |
| RT021962 | 54 | MRM | T2N3M0 | Right：C+S+I | 42.6Gy/16Fx | IMRT |
| RT021967 | 50 | MRM | cT2N1M0/ypT1N1M0 | Right：C+S+I | 46Gy/23Fx | IMRT |
| RT022468 | 51 | MRM | T1N1M0 | Left：C+S+I | 50Gy/25Fx | IMRT |
| RT022478 | 23 | MRM | T2N2M0 | Right：C+S+I | 50Gy/25Fx | IMRT |
| RT022546 | 56 | MRM | T2N1M0 | Left：C+S+I | 50Gy/25Fx | IMRT |
| RT022894 | 52 | MRM | ypT0N0M0 | Right：C+S+I | 50Gy/25Fx | IMRT |
| RT022902 | 56 | MRM | ypT2N1M0 | Left：C+S+I | 50Gy/25Fx | IMRT |
| RT023035 | 53 | MRM | T2N2M0 | Right：C+S+I | 42.6Gy/16Fx | IMRT |
| RT023054 | 60 | MRM | T2N1M0 | Right：C+S+I | 50Gy/25Fx | IMRT |
| RT024652 | 51 | MRM | T2N1M0 | Right：C+S+I | 50Gy/25Fx | IMRT |
| RT025109 | 38 | MRM | T1N2M0 | Right：C+S+I | 50Gy/25Fx | IMRT |
| RT025915 | 50 | MRM | T1N2M0 | Right：C+S+I | 50Gy/25Fx | IMRT |
| RT025912 | 56 | MRM | T1N2M0 | Left：C+S+I | 42.56 Gy/16Fx | IMRT |
| RT026018 | 38 | MRM | pT2N1M0 IIB | Right：C+S+I | 50Gy/25Fx | IMRT |
| RT026627 | 54 | MRM | cT2N1M0/ypT1aN1M0 | Left：C+S+I | 50Gy/25Fx | IMRT |
| RT026620 | 42 | MRM | cTxN1M0/ypT2N0M0 | Right：C+S+I | 50Gy/25Fx | IMRT |
| RT026640 | 45 | MRM | T1N1M0 | Left：C+S+I | 50Gy/25Fx | IMRT |
| RT027183 | 60 | MRM | pT2N2M0 | Left：C+S+I | 42.56 Gy/16Fx | IMRT |
| RT027360 | 45 | MRM | pT2N1M0 | Left：C+S+I | 50Gy/25Fx | IMRT |
| RT027573 | 56 | MRM | T2N1M0 | Right：C+S+I | 50Gy/25Fx | IMRT |
| RT027440 | 48 | MRM | pT2N1M0 | Left：C+S+I | 50Gy/25Fx | IMRT |
| RT027675 | 50 | MRM | T2N1M0 | Left：C+S+I | 42.56 Gy/16Fx | IMRT |
| RT028177 | 64 | MRM | T3N1M0 | Right：C+S+I | 50Gy/25Fx+10Gy/5Fx | SBRT |
| RT028181 | 62 | MRM | T2N1M0 | Right：C+S+I | 42.56 Gy/16Fx | IMRT |
| RT028406 | 48 | MRM | T2N1M0 | Right：C+S+I | 50Gy/25FX | IMRT |
| RT028429 | 54 | MRM | T1N2M0 | Left：C+S+I | 50Gy/25FX | IMRT |
| RT022488 | 50 | MRM | T1N1M0 | Left：C+S+I | 50Gy/25FX | IMRT |
| RT027801 | 66 | MRM | T2N1M0 | Right：C+S+I | 50Gy/25Fx | IMRT |
| RT028660 | 59 | MRM | T1N1M0 | Right：C+S+I | 50Gy/25Fx | IMRT |
| RT028550 | 49 | MRM | T2N2M0 | Right：C+S+I | 50Gy/25Fx | IMRT |
| RT028661 | 67 | MRM | cT3N3M0/ypT1N3M0 | Right：C+S+I | 50Gy/25Fx | IMRT |
| RT028962 | 57 | MRM | TxN1M0 | Right：C+S+I | 50Gy/25Fx | IMRT |
| RT028901 | 43 | MRM | T1N1M0 | Left：C+S+I | 50Gy/25Fx | IMRT |
| RT029137 | 57 | MRM | T1N1M0 | Right：C+S+I | 50Gy/25Fx | IMRT |
| RT029328 | 50 | MRM | T2N2M0 | Right：C+S+I | 42.56 Gy/16Fx | IMRT |
| RT029936 | 68 | MRM | T1N1M0 | Left：C+S+I | 50Gy/25Fx | IMRT |
| RT029789 | 25 | MRM | ypT0N1M0 | Left：C+S+I | 50Gy/25Fx | IMRT |
| RT030257 | 46 | MRM | T2N1M0 | Left：C+S+I | 50Gy/25Fx | IMRT |
| RT030871 | 63 | MRM | pT2N3M0 | Left：C+S+I | 42.56Gy/16Fx | IMRT |
| RT031017 | 56 | MRM | pT1N3bM0 | Left：C+S+I | 50Gy/25Fx | IMRT |
| RT030937 | 67 | MRM | T2N3M0 | Left：C+S+I | 42.56Gy/16Fx | IMRT |
| RT031238 | 53 | MRM | T2N3bM0 | Right：C+S+I | 50Gy/25Fx | IMRT |
| RT031320 | 66 | MRM | T2N1M0 | Right：C+S+I | 42.56Gy/16Fx | IMRT |
| RT031029 | 40 | MRM | pT1N2M0 | Left：C+S+I | 50Gy/25Fx, | IMRT |
| RT031324 | 45 | MRM | T2N3M0 | Right：C+S+I | 42.56Gy/16Fx | IMRT |
| RT031478 | 39 | MRM | T4cN3bM0/ypT3N0M0 | Right：C+S+I | 50Gy/25Fx+10Gy/5Fx | IMRT |
| RT031482 | 70 | MRM | T1N3cM0/ypTN1M0 | Left：C+S+I | 50Gy/25Fx | IMRT |
| RT031675 | 48 | MRM | T2N2M0 | Left：C+S+I | 42.56/16 | IMRT |
| RT031674 | 48 | MRM | T2N1M0 | Left：C+S+I | 42.56/16 | IMRT |
| RT031396 | 75 | MRM | T3N1M0 | Right：C+S+I | 50Gy/25Fx | IMRT |
| RT031870 | 48 | MRM | T2N1M0 | Left：C+S+I | 42.56/16Fx | IMRT |
| RT031936 | 54 | MRM | T2N1M0 | Left：C+S+I | 42.56/15 | IMRT |
| RT032275 | 65 | MRM | T2N1M0 | Right：C+S+I | 50Gy/25Fx | IMRT |
| RT032477 | 55 | MRM | cT4N2M0/ypT3N0M0 | Right：C+S+I | 50Gy/25Fx | IMRT |
| RT032478 | 69 | MRM | T2N1M0 | Right：C+S+I | 42.56/16Fx | IMRT |
| RT032617 | 38 | MRM | T2N2NM0 | Right：C+S+I | 42.56/16Fx | IMRT |
| RT032713 | 60 | MRM | T2N1M0 | Right：C+S+I | 42.56/16Fx | IMRT |
| RT032151 | 64 | MRM | cT4N2M0/ypT0N1M0 | Left：C+S+I | 50/25Gy/25Fx+10Gy/5Fx | IMRT |
| RT033034 | 52 | MRM | T2N3M0 | Left：C+S+I | 42.72Gy/16Fx | IMRT |
| RT032803 | 36 | MRM | T2N1M0 | Left：C+S+I | 42.56Gy/16Fx | IMRT |
| RT032874 | 37 | MRM | T2N1micM0 | Left：C+S+I | 42.56Gy/16Fx | IMRT |
| RT033151 | 46 | MRM | T2N1M0 | Left：C+S+I | 42.56Gy/16Fx | IMRT |
| RT033220 | 58 | MRM | T2N2M0 | Right：C+S+I | 50Gy/25Fx | IMRT |
| RT033235 | 64 | MRM | T1N2M0 | Left：C+S+I | 50Gy/25Fx | IMRT |
| RT033274 | 40 | MRM | T1N1M0 | Right：C+S+I | 42.56Gy/16Fx | IMRT |
| RT033495 | 65 | MRM | T2N3M0 | Right：C+S+I | 50Gy/25Fx | IMRT |
| RT033704 | 66 | MRM | T2N1M0 | Right：C+S+I | 42.56Gy/16Fx | IMRT |
| RT033225 | 48 | MRM | cT2N1M0/ypT1N0M0 | Left：C+S+I | 50Gy/25Fx | IMRT |
| RT033238 | 39 | MRM | T3N0M0 | Left：C+S+I | 42.56Gy/16Fx | IMRT |
| RT034415 | 71 | MRM | TxN1M0 | Right：C+S+I | 50Gy/25Fx | IMRT |
| RT034570 | 59 | MRM | T3N1M0 | Right：C+S+I | 42.56Gy/16Fx+10Gy/5Fx | IMRT |
| RT033377 | 38 | MRM | T2N1M0 | Left：C+S+I | 50Gy/25Fx | IMRT |
| RT033493 | 34 | MRM | T3N2M0/ypT1N0M0 | Left：C+S+I | 50Gy/25Fx | IMRT |
| RT032946 | 40 | MRM | T2N2M0 | Left：C+S+I | 50Gy/25Fx | IMRT |
| RT033592 | 42 | MRM | T1N2M0 | Right：C+S+I | 50Gy/25Fx | IMRT |
| RT034123 | 70 | MRM | T2N2M0 | Left：C+S+I | 42.56GY/16Fx | IMRT |
| RT034775 | 66 | MRM | T1N1M0 | Right：C+S+I | 42.56GY/16Fx | IMRT |
| RT033715 | 45 | MRM | T1N3M0 | Left：C+S+I | 50Gy/25Fx | IMRT |
| RT033678 | 33 | MRM | T2N1M0 | Left：C+S+I | 50Gy/25Fx | IMRT |
| RT035044 | 47 | MRM | T2N1M0 | Left：C+S+I | 50Gy/25FX | IMRT |
| RT033717 | 44 | MRM | T2N1M0 | Left：C+S+I | 42.56Gy/16Fx | IMRT |
| RT035359 | 45 | MRM | pT3N1M0 | Left：C+S+I | 50Gy/25Fx | IMRT |
| RT035625 | 52 | MRM | T2N1M0/ypT0N0M0 | Left：C+S+I | 50Gy/25Fx | IMRT |
| RT035966 | 56 | MRM | T2N1M0 | Right：C+S+I | 50Gy/25FX | IMRT |
| RT035856 | 63 | MRM | pT2N1M0 | Right：C+S+I | 42.5Gy/16Fx | IMRT |
| RT034895 | 38 | MRM | T1N2M0 | Left：C+S+I | 50Gy/25Fx | IMRT |
| RT035566 | 47 | MRM | T2N1M0 | Right：C+S+I | 42.56/16Fx | IMRT |
| RT033154 | 31 | MRM | T2N2M0 | Right：C+S+I | 50Gy/25Fx | IMRT |
| RT035422 | 44 | MRM | T2N1M0 | Left：C+S+I | 42.56Gy/16Fx | IMRT |
| RT035929 | 60 | MRM | pT2N1M0 | Right：C+S+I | 50Gy/25Fx | IMRT |
| RT036110 | 38 | MRM | T2N3M0 | Left：C+S+I | 50Gy/25Fx | IMRT |
| RT035618 | 54 | MRM | T2N1M0 | Right：C+S+I | 50Gy/25Fx | IMRT |
| RT035609 | 43 | MRM | T1N1M0 | Right：C+S+I | 50Gy/25Fx | IMRT |
| RT036766 | 55 | MRM | T2N2M0 | Right：C+S+I | 50Gy/25Fx | IMRT |
| RT037186 | 53 | MRM | T1N1M0 | Left：C+S+I | 42.56Gy/16Fx | IMRT |
| RT035286 | 45 | MRM | T1N1M0 | Left：C+S+I | 42.56/16Fx | IMRT |
| RT036891 | 48 | MRM | T2N3M0 | Right：C+S+I | 50Gy/25Fx | IMRT |
| RT037579 | 71 | MRM | T1N1M0 | Left：C+S+I | 42.56Gy/16Fx | IMRT |
| RT037623 | 56 | MRM | T1N3M0 | Right：C+S+I | 50Gy/25Fx | IMRT |
| RT037492 | 66 | MRM | T2N2M0 | Left：C+S+I | 50Gy/25Fx | IMRT |
| RT037604 | 54 | MRM | T2N2M0 | Right：C+S+I | 42.56/16fx | IMRT |
| RT038429 | 48 | MRM | T2N1M0 | Left：C+S+I | 50Gy/25Fx | IMRT |
| RT037284 | 41 | MRM | T1N1M0 | Right：C+S+I | 50Gy/25Fx | IMRT |
| RT038038 | 51 | MRM | T2N2M0 | Right：C+S+I | 50Gy/25Fx | IMRT |
| RT038311 | 34 | MRM | T2N1M0 | Right：C+S+I | 42.56/16fx | IMRT |
| RT038761 | 53 | MRM | T2N1M0 | Left：C+S+I | 42.56/16fx | IMRT |
| RT038471 | 55 | MRM | T3N1M0 | Left：C+S+I | 42.56/16fx | IMRT |
| RT038891 | 40 | MRM | T1N1M0 | Right：C+S+I | 42.56/16fx | IMRT |
| RT038989 | 46 | MRM | cT2N1M0/ypT1N2M0 | Left：C+S+I | 50Gy/25Fx | IMRT |
| RT038758 | 73 | MRM | T4cN3M0/ypT1N0M0 | Right：C+S+I | 50Gy/25Fx | IMRT |
| RT038878 | 43 | MRM | T1N1M0 | Right：C+S+I | 50Gy/25Fx | IMRT |
| RT039436 | 51 | MRM | T1N1M0 | Left：C+S+I | 42.56/16fx | IMRT |
| RT039536 | 42 | MRM | T1N1M0 | Left：C+S+I | 50Gy/25Fx | IMRT |
| RT039245 | 36 | MRM | T2N3M0 | Left：C+S+I | 50Gy/25Fx | IMRT |
| RT039407 | 58 | MRM | T1N1M0 | Left：C+S+I | 42.56/16fx | IMRT |
| RT039444 | 49 | MRM | T1N2M0 | Right：C+S+I | 42.56/16fx | IMRT |
| RT039928 | 38 | MRM | T3N1M0 | Right：C+S+I | 50Gy/25Fx | IMRT |
| RT039688 | 57 | MRM | T2N2M0 | Right：C+S+I | 50Gy/25Fx | IMRT |
| RT040073 | 35 | MRM | T1N1M0 | Right：C+S+I | 50Gy/25Fx | IMRT |
| RT040223 | 55 | MRM | T2N2M0 | Left：C+S+I | 42.56/16fx | IMRT |
| RT040214 | 58 | MRM | T1N2M0 | Left：C+S+I | 42.56/16fx | IMRT |
| RT040479 | 51 | MRM | ypT2N2M0 | Right：C+S+I | 42.5Gy/16Fx | IMRT |
| RT039814 | 61 | MRM | T2N2M0 | Right：C+S+I | 42.56/16FX | IMRT |
| RT040305 | 53 | MRM | T2N2M0 | Left：C+S+I | 42.56/16fx | IMRT |
| RT040156 | 75 | MRM | T2N1M0 | Right：C+S+I | 42.56Gy/16Fx | IMRT |
| RT040568 | 54 | MRM | ypT2N3M0 | Right：C+S+I | 50Gy/25Fx | IMRT |
| RT039557 | 45 | MRM | cT4N1M0/ypT1N2M0 | Left：C+S+I | 50Gy/25Fx | IMRT |
| RT039819 | 55 | MRM | ypT2N1M0 | Right：C+S+I | 42.56/16fx | IMRT |
| RT040475 | 76 | MRM | T2N1M0 | Right：C+S+I | 42.56/16fx | IMRT |
| RT040995 | 50 | MRM | cT3N2M0/ypT2N2M0 | Left：C+S+I | 50/25Fx | IMRT |
| RT040253 | 72 | MRM | T2N1M0 | Right：C+S+I | 42.56Gy/16Fx | IMRT |
| RT041137 | 56 | MRM | T3N0M0 | Right：C+S+I | 50Gy/25Fx | IMRT |
| RT040722 | 51 | MRM | T1N1M0 | Right：C+S+I | 42.56Gy/16Fx | IMRT |
| RT041473 | 51 | MRM | T2N1M0 | Right：C+S+I | 42.56Gy/16Fx | IMRT |
| RT040963 | 56 | MRM | T1N1M0 | Left：C+S+I | 50Gy/25Fx | IMRT |
| RT041247 | 63 | MRM | T3N1M0/ypT2N0M0 | Right：C+S+I | 50/25Fx | IMRT |
| RT040320 | 51 | MRM | T2N1M0 | Right：C+S+I | 50Gy/25Fx | IMRT |
| RT040599 | 54 | MRM | T2N1M0 | Right：C+S+I | 50/25Fx | IMRT |
| RT040870 | 35 | MRM | T3N2M0 | Right：C+S+I | 50Gy/25Fx | IMRT |
| RT041352 | 33 | MRM | T2N1M0 | Right：C+S+I | 42.56Gy/16Fx | IMRT |
| RT041731 | 48 | MRM | T2N2M0 | Right：C+S+I | 50Gy/25Fx | IMRT |
| RT040670 | 49 | MRM | pT1N2M0 | Left：C+S+I | 50Gy/25Fx | IMRT |
| RT041723 | 42 | MRM | T1N1M0 | Right：C+S+I | 42.56Gy/16Fx | IMRT |
| RT041923 | 57 | MRM | T2N1M0 | Right：C+S+I | 42.56Gy/16Fx | IMRT |
| RT041926 | 52 | MRM | T1N1M0 | Right：C+S+I | 42.56Gy/16Fx | IMRT |
| RT041933 | 49 | MRM | T2N2M0 | Left：C+S+I | 50Gy/25Fx | IMRT |
| RT041934 | 48 | MRM | T2N2M0 | Left：C+S+I | 50Gy/25Fx | IMRT |
| RT041946 | 60 | MRM | T2N1M0 | Right：C+S+I | 42.56Gy/16Fx | IMRT |
| RT040802 | 38 | MRM | T2N1M0 | Left：C+S+I | 50Gy/25Fx | IMRT |
| RT042469 | 72 | MRM | T1N1M0 | Left：C+S+I | 42.56Gy/16Fx | IMRT |
| RT042445 | 69 | MRM | T1N1M0 | Right：C+S+I | 42.56Gy/16Fx | IMRT |
| RT042623 | 44 | MRM | T2N1M0 | Left：C+S+I | 42.56Gy/16Fx | IMRT |
| RT041733 | 54 | MRM | T2N1M0 | Left：C+S+I | 50Gy/25Fx | IMRT |
| RT041929 | 37 | MRM | T2N1M0 | Right：C+S+I | 50Gy/25Fx | IMRT |
| RT042912 | 48 | MRM | T3N1M0/ypT0N3M0 | Left：C+S+I | 42.56Gy/16Fx | IMRT |
| RT042914 | 66 | MRM | T2N1M0 | Right：C+S+I | 50Gy/25Fx | IMRT |
| RT042148 | 40 | MRM | T2N2M0 | Right：C+S+I | 50Gy/25Fx | IMRT |
| RT041762 | 60 | MRM | T2N2M0 | Left：C+S+I | 42.56Gy/16Fx | IMRT |
| RT043020 | 57 | MRM | cT2N0M0/ypT0N1M0 | Right：C+S+I | 42.56Gy/16Fx | IMRT |
| RT043133 | 40 | MRM | cT2N1M0/ypT1N1M0 | Left：C+S+I | 50Gy/25Fx | IMRT |
| RT041423 | 57 | MRM | T2N1M0 | Right：C+S+I | 42.56Gy/16Fx | IMRT |
| RT041391 | 60 | MRM | T1N1M0 | Left：C+S+I | 42.56Gy/16Fx | IMRT |
| RT043390 | 59 | MRM | T3N0M0 | Right：C+S+I | 50Gy/25Fx | IMRT |
| RT043428 | 54 | MRM | T1N1M0 | Right：C+S+I | 50Gy/25Fx | IMRT |
| RT043074 | 40 | MRM | cT2N1M0/ypT2N3M0 | Right：C+S+I | 50Gy/25Fx | IMRT |
| RT043052 | 43 | MRM | ypT2N3M0 | Left：C+S+I | 50Gy/25Fx | IMRT |
| RT042858 | 62 | MRM | T1N2M0 | Right：C+S+I | 50Gy/25Fx | IMRT |
| RT043306 | 50 | MRM | cT2N1M0/ypT1N2M0 | Right：C+S+I | 50Gy/25Fx | IMRT |
| RT043928 | 27 | MRM | T3N3M0 | Right：C+S+I | 42.56Gy/16Fx | IMRT |
| RT043063 | 53 | MRM | T2N2M0 | Left：C+S+I | 42.56Gy/16Fx | IMRT |
| RT043460 | 52 | MRM | T2N1M0 | Right：C+S+I | 42.56Gy/16Fx | IMRT |
