# Supplementary material for: Geometric and dosimetric evaluation of deep learning based auto‐segmentation for clinical target volume on breast cancer
Source: J Appl Clin Med Phys. 2023 Mar 15;24(7):e13951. doi: 10.1002/acm2.13951 (PMC10338811; doi:10.1002/acm2.13951)
Supplement: Supplementary file 2 — Fig. 2 The CNN model used in our study. (a) The architecture of the VB‐based U‐net for target segmentation model. (b)The two process (coarse segmentation and fine segmentation) of this model. (c) The details of down block, up block, and bottleneck used in this U‐net. [file ACM2-24-e13951-s002.docx]

The VB-Net was formed of an Input Block, four Down Blocks, four Up Blocks, and an Output Block. The shape of the output feature of each block is shown in Figure 2. The Input Block was formed of a 3D convolution layer with a kernel size of [5, 5, 5] and a padding of [2, 2, 2], a BatchNorm layer, and a Relu activation layer. The input of the Input Block was a 3D image patch of [B, 1, 256, 256, 64], and the output was a feature of [B, 16, 256, 256, 64], where B represents the batch size, which is 4 in this paper.

The Down Block was composed of a 3D convolution layer with a kernel size of [3, 3, 3] and a stride of [2, 2, 2], a BatchNorm layer, a Relu activation layer, and two bottlenecks. The Down Block enlarged the number of feature channels and compressed the feature size.

The Up Block consists of a deconvolution block, a convolution block, and two bottlenecks. The deconvolution block was composed of a 3D deconvolution layer with a kernel size of [3, 3, 3] and a stride of [2, 2, 2], a BatchNorm layer, and a Relu activation layer. The Up Block compressed the number of feature channels and expanded the size of features. Take the Up Block before the Output Block as an example. The output features with the shape of [B, 32, 128, 128, 32] from the previous Up Block have carried out deconvolution operation in the deconvolution block to get the feature with the shape of [B, 16, 256, 256, 64]. Then this feature was concatenated with the Input Block's feature as the following convolution block's input. Finally, the output of the Up Block was the feature with a shape of [B, 16, 256, 256, 64].

The Bottleneck Block was composed of three convolution blocks. Each convolution block comprised a 3D convolution layer, a BatchNorm layer, and a Relu activation layer. In the first convolution block, the kernel size of the 3D convolution layer was [1, 1, 1], and the number of output channels was 1/4 of the number of input channels. In the second convolution block, the kernel size of the 3D convolution layer was [3, 3, 3], and the output of the convolution core was consistent with the input channel. In the third convolution block, the kernel size of the 3D convolution layer was [1, 1, 1], and the number of output channels was four times the number of input channels.

The Output Block was composed of a 3D convolution layer with a kernel size of [3, 3, 3], a BatchNorm layer, and a Relu activation layer.


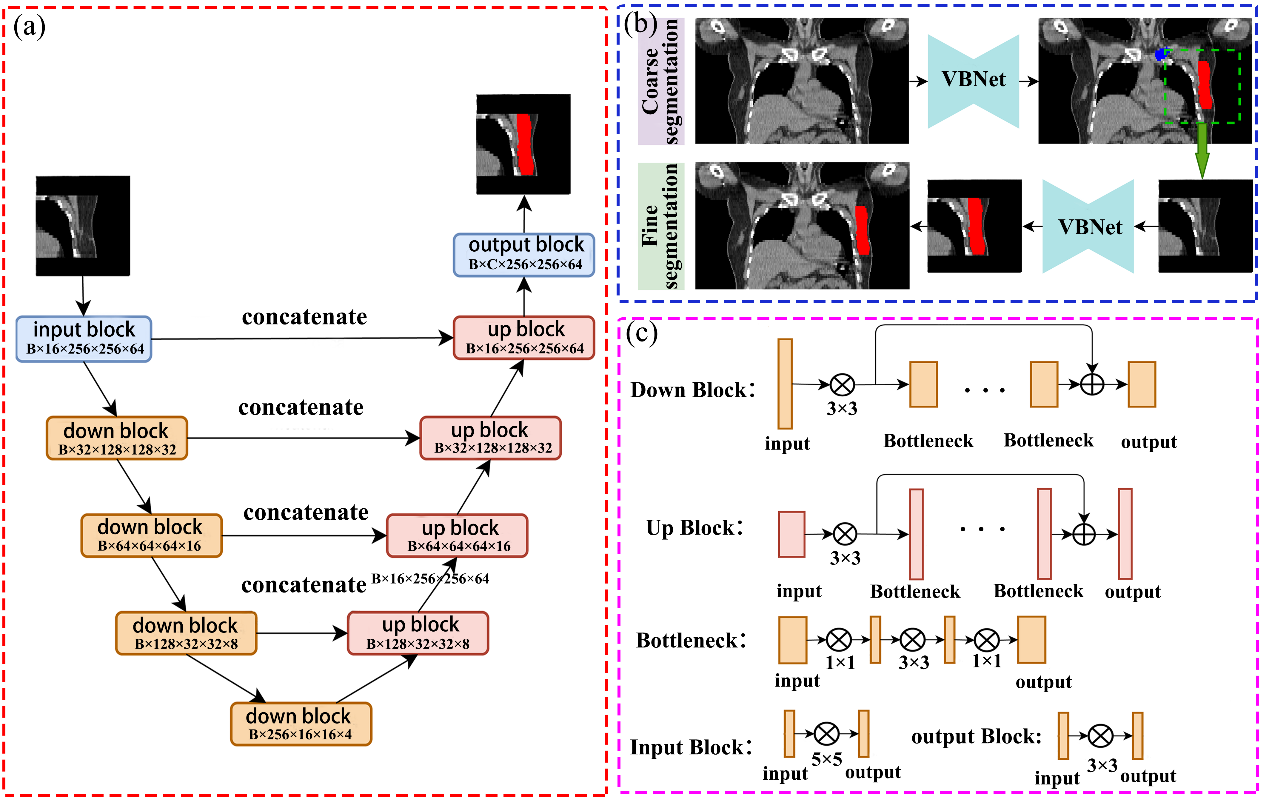


**Fig. 2 The CNN model used in our study. (a) The architecture of the VB-based U-net for target segmentation model. (b)The two process (coarse segmentation and fine segmentation) of this model. (c) The details of down block, up block and bottleneck used in this U-net.**
