# Supplementary material for: Geometric and dosimetric evaluation of deep learning based auto‐segmentation for clinical target volume on breast cancer
Source: J Appl Clin Med Phys. 2023 Mar 15;24(7):e13951. doi: 10.1002/acm2.13951 (PMC10338811; doi:10.1002/acm2.13951)
Supplement: Supplementary file 3 — Supporting Information [file ACM2-24-e13951-s001.docx]

| DICE |  |  |  |  |  |  |  |  |  |
| --- | --- | --- | --- | --- | --- | --- | --- | --- | --- |
| Patient_id | CTV_ALN | CTV_CW | CTV_IMN | CTV_SCN | PTV_ALN | PTV_CW_EVA | PTV_IMN | PTV_SCN | PTVall |
| RT014017 | 0.694591 | 0.558083 | 0.553148 | 0.740756 | 0.77574 | 0.781081 | 0.59414 | 0.785096 | 0.793359 |
| RT014099 | 0.810458 | 0.766875 | 0.521815 | 0.637217 | 0.876302 | 0.859645 | 0.700481 | 0.67705 | 0.847171 |
| RT014263 | 0.176635 | 0.770954 | 0.425283 | 0.729209 | 0.296454 | 0.839499 | 0.57138 | 0.766766 | 0.805675 |
| RT014338 | 0.786517 | 0.70223 | 0.562806 | 0.56601 | 0.818069 | 0.836613 | 0.608961 | 0.605235 | 0.823236 |
| RT014432 | 0.866591 | 0.710129 | 0.371806 | 0.739198 | 0.892749 | 0.814955 | 0.480773 | 0.786107 | 0.823528 |
| RT014482 | 0.864529 | 0.803041 | 0.639433 | 0.716065 | 0.891546 | 0.861454 | 0.624131 | 0.738155 | 0.852035 |
| RT014753 | 0.854313 | 0.737616 | 0.506667 | 0.672838 | 0.885206 | 0.825005 | 0.601066 | 0.660184 | 0.819695 |
| RT014765 | 0.783861 | 0.731927 | 0.533333 | 0.589152 | 0.817243 | 0.834253 | 0.625904 | 0.660647 | 0.829252 |
| RT014819 | 0.868547 | 0.592428 | 0.480707 | 0.717506 | 0.875949 | 0.773599 | 0.624245 | 0.786693 | 0.815142 |
| RT014917 | 0.773882 | 0.725409 | 0.32049 | 0.695375 | 0.845371 | 0.803371 | 0.547849 | 0.762838 | 0.80464 |
| RT014990 | 0.626189 | 0.714607 | 0.413667 | 0.670915 | 0.670151 | 0.817942 | 0.447958 | 0.668215 | 0.802277 |
| RT015074 | 0.711251 | 0.694937 | 0.442774 | 0.586381 | 0.742801 | 0.782141 | 0.561901 | 0.60153 | 0.788721 |
| RT015121 | 0.850665 | 0.728936 | 0.660465 | 0.673011 | 0.883338 | 0.827286 | 0.663715 | 0.73779 | 0.833079 |
| RT015125 | 0.552157 | 0.699615 | 0.518837 | 0.607276 | 0.615331 | 0.855196 | 0.594781 | 0.618035 | 0.819584 |
| RT015385 | 0.78769 | 0.694898 | 0.567326 | 0.671193 | 0.824767 | 0.783966 | 0.62478 | 0.720576 | 0.796183 |
| RT015387 | 0.790926 | 0.695003 | 0.414773 | 0.749567 | 0.816512 | 0.765423 | 0.558809 | 0.807463 | 0.782724 |
| RT015389 | 0.836707 | 0.690059 | 0.699521 | 0.756779 | 0.877548 | 0.82992 | 0.744335 | 0.772172 | 0.841312 |
| RT015442 | 0.811541 | 0.662203 | 0.589865 | 0.67233 | 0.855413 | 0.848068 | 0.655992 | 0.746438 | 0.852622 |
| RT015495 | 0.761146 | 0.684773 | 0.227175 | 0.524532 | 0.790447 | 0.860241 | 0.443201 | 0.568931 | 0.804763 |
| RT015573 | 0.82248 | 0.605955 | 0.460381 | 0.625575 | 0.840259 | 0.787485 | 0.564152 | 0.691372 | 0.801301 |
| RT015833 | 0.743383 | 0.743605 | 0.5299 | 0.753742 | 0.781861 | 0.830218 | 0.626879 | 0.82929 | 0.855458 |
| RT015843 | 0.84564 | 0.670849 | 0.396567 | 0.763931 | 0.87199 | 0.856394 | 0.519294 | 0.800897 | 0.865236 |
| RT016128 | 0.840912 | 0.731125 | 0.620629 | 0.731976 | 0.888332 | 0.829885 | 0.673255 | 0.812885 | 0.849107 |
| RT016140 | 0.798789 | 0.774177 | 0.618248 | 0.716637 | 0.839155 | 0.850849 | 0.618374 | 0.799144 | 0.848069 |
| RT016141 | 0.857777 | 0.787731 | 0.511651 | 0.788582 | 0.880471 | 0.850046 | 0.585484 | 0.818113 | 0.852872 |
| RT016160 | 0.789176 | 0.753417 | 0.543551 | 0.789779 | 0.831922 | 0.847849 | 0.634501 | 0.827994 | 0.857612 |
| RT016223 | 0.829602 | 0.835059 | 0.674808 | 0.770367 | 0.851367 | 0.871342 | 0.683844 | 0.850291 | 0.878653 |
| RT016324 | 0.832801 | 0.723808 | 0.501054 | 0.717713 | 0.84819 | 0.823157 | 0.528992 | 0.778769 | 0.82934 |
| RT016326 | 0.816114 | 0.691691 | 0.565265 | 0.783635 | 0.849029 | 0.834353 | 0.625341 | 0.833467 | 0.852405 |
| RT016334 | 0.807304 | 0.804274 | 0.448313 | 0.757378 | 0.863416 | 0.854928 | 0.58928 | 0.819839 | 0.855089 |
| RT016336 | 0.867628 | 0.801046 | 0.471387 | 0.669922 | 0.897136 | 0.860357 | 0.583847 | 0.794227 | 0.866581 |
| RT016478 | 0.82419 | 0.711422 | 0.524395 | 0.722927 | 0.861859 | 0.779458 | 0.519829 | 0.800604 | 0.799622 |
| RT016541 | 0.713256 | 0.780525 | 0.519723 | 0.79914 | 0.784334 | 0.861714 | 0.585087 | 0.848668 | 0.861804 |
| RT016557 | 0.781095 | 0.796291 | 0.622733 | 0.734516 | 0.798411 | 0.855269 | 0.583446 | 0.777586 | 0.84668 |
| RT016562 | 0.833407 | 0.702115 | 0.605141 | 0.719779 | 0.864691 | 0.789691 | 0.652155 | 0.765407 | 0.816057 |
| RT016705 | 0.776637 | 0.747038 | 0.597777 | 0.791742 | 0.803067 | 0.842801 | 0.642648 | 0.801613 | 0.848803 |
| RT016746 | 0.789122 | 0.796629 | 0.406627 | 0.751853 | 0.828612 | 0.866106 | 0.497815 | 0.825903 | 0.859053 |
| RT016804 | 0.730081 | 0.761775 | 0.536496 | 0.804415 | 0.778971 | 0.831127 | 0.588937 | 0.852327 | 0.847804 |
| RT016814 | 0.753344 | 0.657316 | 0.714772 | 0.675132 | 0.806248 | 0.754945 | 0.707361 | 0.763856 | 0.799061 |
| RT017291 | 0.840108 | 0.705767 | 0.283218 | 0.738906 | 0.897949 | 0.815747 | 0.230154 | 0.802292 | 0.820905 |
| RT017292 | 0.858119 | 0.71184 | 0.641212 | 0.747479 | 0.890787 | 0.797797 | 0.683561 | 0.694605 | 0.811955 |
| RT017299 | 0.761572 | 0.813586 | 0.474811 | 0.805577 | 0.799424 | 0.878578 | 0.609119 | 0.848441 | 0.870964 |
| RT017301 | 0.792849 | 0.79471 | 0.496018 | 0.735875 | 0.826484 | 0.864806 | 0.571954 | 0.805326 | 0.861131 |
| RT017494 | 0.80496 | 0.742308 | 0.507338 | 0.671434 | 0.829614 | 0.832464 | 0.579168 | 0.730689 | 0.826934 |
| RT040223 | 0.741672 | 0.804519 | 0.467831 | 0.829246 | 0.836372 | 0.849226 | 0.557408 | 0.87973 | 0.865667 |
| RT041723 | 0.767851 | 0.564758 | 0.583228 | 0.768295 | 0.81175 | 0.749197 | 0.690081 | 0.876518 | 0.806818 |
| RT041731 | 0.74786 | 0.752055 | 0.503463 | 0.708638 | 0.790375 | 0.844706 | 0.631545 | 0.824874 | 0.871219 |
| RT043133 | 0.829501 | 0.771561 | 0.368838 | 0.765772 | 0.87539 | 0.839404 | 0.567366 | 0.846531 | 0.867513 |
| HD |  |  |  |  |  |  |  |  |  |
| Patient_id | CTV_ALN | CTV_CW | CTV_IMN | CTV_SCN | PTV_ALN | PTV_CW_EVA | PTV_IMN | PTV_SCN | PTVall |
| RT014017 | 0.694591 | 0.558083 | 0.553148 | 0.740756 | 0.77574 | 0.781081 | 0.59414 | 0.785096 | 0.793359 |
| RT014099 | 16.63316 | 22.22886 | 15.2016 | 23.16665 | 14.56439 | 21.10479 | 16.13427 | 24.16994 | 24.16994 |
| RT014263 | 45.70942 | 20.08418 | 30.20924 | 16.49214 | 46.81493 | 19.27126 | 31.02546 | 18.089 | 25.02324 |
| RT014338 | 11.50714 | 31.49411 | 43.58002 | 40.81464 | 12.58355 | 31.41166 | 44.92775 | 39.86649 | 22.3215 |
| RT014432 | 8.938764 | 47.12457 | 40.13197 | 11.37434 | 9.151563 | 27.6209 | 40.13197 | 11.97814 | 27.23534 |
| RT014482 | 6.394278 | 12.50695 | 35.65768 | 11.63786 | 7.738281 | 13.87164 | 36.52116 | 12.78856 | 15 |
| RT014753 | 6.208898 | 25.2577 | 34.22 | 20.95939 | 7.027855 | 23.6794 | 34.31885 | 25.69496 | 25.69496 |
| RT014765 | 12.02573 | 20.07525 | 33.97169 | 21.89341 | 12.67795 | 19.09435 | 34.47868 | 22.47996 | 22.47996 |
| RT014819 | 7.577225 | 35.72385 | 28.78475 | 10.12883 | 8.466206 | 34.96903 | 29.781 | 10 | 34.96903 |
| RT014917 | 6.256162 | 21.63439 | 26.25192 | 5.664063 | 6.332614 | 21.02524 | 26.61601 | 6.230469 | 21.12419 |
| RT014990 | 30.48059 | 25.02924 | 40.89063 | 20.75769 | 31.11692 | 23.23265 | 45.3217 | 25.74895 | 25.74895 |
| RT015074 | 31.16228 | 33.08258 | 50.1054 | 141.9135 | 29.48782 | 31.92894 | 50.05273 | 141.6902 | 31.92894 |
| RT015121 | 9.848077 | 24.29805 | 31.40063 | 17.26584 | 9.13715 | 20.37362 | 38.94784 | 17.77936 | 20.37362 |
| RT015125 | 35.33052 | 19.54095 | 33.62188 | 26.52532 | 36.59281 | 17.6845 | 35.11999 | 31.34737 | 31.34737 |
| RT015385 | 12.29632 | 26.61326 | 28.06609 | 17.885 | 15.14137 | 27.47913 | 29.84102 | 19.81743 | 22.59618 |
| RT015387 | 15.35002 | 37.62766 | 41.32769 | 12.24958 | 15.77663 | 35.54058 | 42.85771 | 12.4645 | 35.54058 |
| RT015389 | 12.63333 | 16.70128 | 21.4436 | 14.10798 | 11.393 | 15.44034 | 22.44845 | 14.02323 | 15.44034 |
| RT015442 | 9.10748 | 28.76711 | 38.32963 | 14.69328 | 10.8496 | 20.55148 | 40.00631 | 12.97103 | 16.80888 |
| RT015495 | 14.00737 | 16.52441 | 45.82302 | 45.55282 | 15.40245 | 17.03038 | 47.35656 | 45.05297 | 43.85973 |
| RT015573 | 11.56294 | 33.30854 | 30.10638 | 19.74961 | 13.01957 | 32.56011 | 30.02131 | 19.74961 | 32.56011 |
| RT015833 | 23.81006 | 24.83159 | 29.27979 | 10.08085 | 24.63218 | 23.72866 | 31.0109 | 9.818917 | 23.72866 |
| RT015843 | 6.837812 | 22.41607 | 31.76127 | 10.42604 | 7.51969 | 21.27068 | 32.42547 | 10.83797 | 16.18888 |
| RT016128 | 8.605437 | 15.5595 | 52.83219 | 13.50132 | 9.088354 | 51.62186 | 109.2356 | 13.6354 | 24.60173 |
| RT016140 | 10.27277 | 17.8058 | 42.54139 | 26.76249 | 11.05076 | 16.12509 | 317.91 | 13.6149 | 16.12509 |
| RT016141 | 9.605424 | 17.57526 | 30.26528 | 12.34988 | 9.940389 | 15.53404 | 32.32889 | 10 | 13.24619 |
| RT016160 | 17.60043 | 23.8449 | 37.23879 | 11.97469 | 17.0521 | 14.95939 | 37.68746 | 9.903644 | 15 |
| RT016223 | 17.1615 | 15.71408 | 29.60578 | 16.05108 | 15.18382 | 14.74767 | 32.12249 | 9.705189 | 15 |
| RT016324 | 10.96292 | 22.7224 | 41.55216 | 15.59796 | 12.0493 | 21.9817 | 43.14492 | 14.58207 | 15.59796 |
| RT016326 | 14.92655 | 22.46633 | 42.48518 | 10.33541 | 12.91679 | 22.67785 | 44.0459 | 10.85053 | 31.6497 |
| RT016334 | 12.25149 | 15.53326 | 33.55578 | 11.83551 | 11.18255 | 16.28125 | 35.08843 | 10.78342 | 15.04169 |
| RT016336 | 9.886641 | 18.26571 | 27.4203 | 16.62585 | 10.97095 | 16.98437 | 25.69217 | 11.60037 | 15.1581 |
| RT016478 | 10 | 57.55595 | 49.44717 | 13.36391 | 10.75727 | 56.87972 | 56.81112 | 11.87901 | 56.87972 |
| RT016541 | 30.26762 | 130.503 | 175.1241 | 8.597642 | 30.2864 | 100.574 | 169.2126 | 7.763555 | 29.85147 |
| RT016557 | 21.89598 | 22.44716 | 43.37371 | 75.5971 | 21.55851 | 38.74374 | 289.1891 | 15.01393 | 29.18521 |
| RT016562 | 10.57006 | 168.8136 | 189.6963 | 19.55822 | 8.954329 | 63.25456 | 191.5477 | 21.4181 | 63.25456 |
| RT016705 | 35.00736 | 11.15627 | 37.93527 | 12.12398 | 33.50587 | 11.96582 | 40.27367 | 14.24331 | 15 |
| RT016746 | 9.527344 | 13.81491 | 60.53921 | 7.281176 | 10.58594 | 13.6927 | 60.34454 | 8.152482 | 13.6927 |
| RT016804 | 23.75627 | 37.01598 | 35.47389 | 8.199481 | 23.75627 | 35.3601 | 41.62835 | 8.546875 | 35.3601 |
| RT016814 | 13.01957 | 36.79697 | 26.82743 | 13.59615 | 14.44651 | 35.08914 | 27.90229 | 15.47236 | 35.08914 |
| RT017291 | 15.578 | 16.76091 | 66.13154 | 12.77139 | 10.71625 | 17.2641 | 65.84615 | 12.816 | 24.78041 |
| RT017292 | 6.218164 | 37.20113 | 36.5481 | 13.14682 | 6.307447 | 36.17905 | 37.30265 | 20.99857 | 36.17905 |
| RT017299 | 23.47565 | 12.57299 | 28.47765 | 8.210755 | 22.64395 | 11.81186 | 29.32227 | 9.148194 | 12.17413 |
| RT017301 | 12.12305 | 20.0068 | 40.63857 | 11.88446 | 11.54216 | 19.65686 | 42.09249 | 10.93045 | 19.65686 |
| RT017494 | 17.38132 | 26.49736 | 30.49789 | 16.70982 | 16.85342 | 26.90349 | 30.25992 | 17.69051 | 21.10669 |
| RT040223 | 11.84882 | 15.03176 | 78.37442 | 12.00872 | 10.11176 | 15.15811 | 67.55143 | 11.68674 | 15.15811 |
| RT041723 | 30.02608 | 44.91354 | 48.2965 | 10.05433 | 30.02608 | 44.34728 | 43.01872 | 10.05433 | 44.34728 |
| RT041731 | 26.66141 | 17.82059 | 40.98662 | 11.35564 | 26.66141 | 18.00692 | 40.75327 | 10.9183 | 18.00692 |
| RT043133 | 10.97121 | 22.40172 | 143.097 | 8.525475 | 10.97121 | 21.68321 | 143.4994 | 8.525475 | 21.68321 |
| MDA |  |  |  |  |  |  |  |  |  |
| Patient_id | CTV_ALN | CTV_CW | CTV_IMN | CTV_SCN | PTV_ALN | PTV_CW_EVA | PTV_IMN | PTV_SCN | PTVall |
| RT014017 | 1.437167 | 1.826503 | 2.400028 | 0.935701 | 1.012852 | 0.938773 | 3.46964 | 0.776575 | 0.849606 |
| RT014099 | 0.744669 | 0.802179 | 2.094434 | 2.143253 | 0.515713 | 0.511717 | 1.23085 | 1.997403 | 0.729985 |
| RT014263 | 14.01027 | 0.991377 | 3.801621 | 1.023235 | 11.36242 | 0.733501 | 2.91502 | 0.911495 | 1.115907 |
| RT014338 | 0.842575 | 1.101471 | 3.871727 | 2.306085 | 0.704285 | 0.667862 | 3.852609 | 3.037456 | 0.815659 |
| RT014432 | 0.344579 | 1.195318 | 7.806082 | 0.846987 | 0.286832 | 0.866861 | 8.169763 | 0.74851 | 0.785643 |
| RT014482 | 0.350276 | 0.621854 | 2.672708 | 1.20418 | 0.285486 | 0.466084 | 2.679865 | 1.068833 | 0.511777 |
| RT014753 | 0.315181 | 1.078181 | 2.966639 | 1.851305 | 0.252635 | 0.844869 | 2.89144 | 2.313479 | 1.025862 |
| RT014765 | 0.85469 | 0.835454 | 3.391107 | 2.638243 | 0.737094 | 0.58948 | 3.350355 | 2.17507 | 0.853428 |
| RT014819 | 0.372558 | 1.673229 | 3.638133 | 1.020541 | 0.339848 | 1.223031 | 2.933963 | 0.82245 | 0.914158 |
| RT014917 | 0.446732 | 1.072974 | 4.549871 | 0.625301 | 0.287198 | 0.85939 | 3.226085 | 0.510811 | 0.65833 |
| RT014990 | 3.126865 | 1.252932 | 7.503311 | 1.857326 | 2.724446 | 0.897862 | 8.012659 | 2.348223 | 1.123981 |
| RT015074 | 2.223014 | 1.704465 | 8.375102 | 10.39428 | 2.061314 | 1.372809 | 8.134733 | 13.82032 | 1.298613 |
| RT015121 | 0.457411 | 1.147721 | 2.219215 | 1.501341 | 0.351326 | 0.731533 | 3.019035 | 1.273909 | 0.751521 |
| RT015125 | 4.072798 | 0.976956 | 4.729907 | 3.320326 | 3.555018 | 0.511162 | 4.574457 | 3.769645 | 1.214516 |
| RT015385 | 0.709492 | 1.904524 | 2.344872 | 1.492931 | 0.60121 | 1.496906 | 2.227929 | 1.340776 | 1.038422 |
| RT015387 | 0.741993 | 2.241119 | 5.71107 | 0.887043 | 0.689837 | 1.942456 | 4.873949 | 0.673802 | 1.52202 |
| RT015389 | 0.476513 | 1.045283 | 1.281883 | 0.770209 | 0.385113 | 0.595607 | 1.269624 | 0.898002 | 0.607073 |
| RT015442 | 0.585857 | 1.297078 | 3.146885 | 1.411792 | 0.456455 | 0.719012 | 3.337361 | 1.082523 | 0.609334 |
| RT015495 | 0.984728 | 0.909501 | 6.796049 | 5.725029 | 0.87998 | 0.495045 | 4.491381 | 5.587727 | 1.734201 |
| RT015573 | 0.527081 | 1.876797 | 4.426082 | 2.274325 | 0.503707 | 1.210202 | 4.529898 | 1.967443 | 1.155797 |
| RT015833 | 1.092669 | 1.069404 | 2.402485 | 0.78318 | 1.015424 | 0.767123 | 2.158939 | 0.562333 | 0.554264 |
| RT015843 | 0.375294 | 0.962488 | 5.018163 | 0.839705 | 0.326918 | 0.498414 | 4.378166 | 0.735516 | 0.403133 |
| RT016128 | 0.46522 | 0.95858 | 2.923807 | 1.011054 | 0.32507 | 0.735517 | 3.761221 | 0.695812 | 0.56479 |
| RT016140 | 0.763863 | 0.766791 | 2.264566 | 1.30053 | 0.612405 | 0.547413 | 6.924193 | 0.729034 | 0.521621 |
| RT016141 | 0.319504 | 0.864198 | 2.684608 | 0.67267 | 0.286948 | 0.612578 | 2.541842 | 0.624592 | 0.501936 |
| RT016160 | 0.970568 | 0.82735 | 2.875852 | 0.687289 | 0.87332 | 0.502968 | 2.933107 | 0.555996 | 0.493614 |
| RT016223 | 0.726733 | 0.588468 | 2.094918 | 0.903128 | 0.662537 | 0.468705 | 2.118204 | 0.429386 | 0.376033 |
| RT016324 | 0.615303 | 1.125164 | 5.372316 | 1.154514 | 0.561665 | 0.77715 | 5.105901 | 0.897598 | 0.709731 |
| RT016326 | 0.708042 | 1.123445 | 4.615147 | 0.640396 | 0.566211 | 0.77068 | 4.921998 | 0.537647 | 0.646814 |
| RT016334 | 0.74158 | 0.708326 | 3.806824 | 0.912282 | 0.512745 | 0.583098 | 3.394695 | 0.68984 | 0.48576 |
| RT016336 | 0.342887 | 0.608237 | 3.328057 | 1.279801 | 0.283881 | 0.425347 | 2.760906 | 0.766066 | 0.406955 |
| RT016478 | 0.550821 | 2.658365 | 4.43094 | 1.067889 | 0.444649 | 2.242994 | 6.032782 | 0.733091 | 1.852304 |
| RT016541 | 2.209351 | 1.242139 | 9.876067 | 0.530182 | 1.765869 | 1.078461 | 10.75069 | 0.406914 | 0.720196 |
| RT016557 | 1.32823 | 0.983618 | 2.657982 | 2.560983 | 1.248007 | 0.832825 | 7.48311 | 0.84419 | 0.597811 |
| RT016562 | 0.449835 | 3.539493 | 10.98643 | 1.125497 | 0.374827 | 2.138746 | 11.7697 | 0.934555 | 1.333319 |
| RT016705 | 1.412093 | 0.863708 | 3.219801 | 0.69628 | 1.360266 | 0.570311 | 3.267203 | 0.732383 | 0.553663 |
| RT016746 | 0.628916 | 0.56199 | 10.0157 | 0.705465 | 0.509863 | 0.449102 | 8.772561 | 0.541471 | 0.406456 |
| RT016804 | 1.844499 | 1.905577 | 3.58464 | 0.522976 | 1.520744 | 1.580525 | 3.216828 | 0.421648 | 0.879327 |
| RT016814 | 1.089085 | 2.016447 | 1.435636 | 1.228871 | 0.903152 | 1.620579 | 1.632034 | 0.92468 | 1.115051 |
| RT017291 | 0.544079 | 1.19518 | 14.52163 | 0.880901 | 0.339837 | 0.852145 | 20.87106 | 0.637665 | 0.778137 |
| RT017292 | 0.39373 | 1.616444 | 3.285149 | 0.931145 | 0.307995 | 1.405095 | 3.318524 | 1.770493 | 1.122919 |
| RT017299 | 1.456914 | 0.55193 | 2.846001 | 0.467411 | 1.348797 | 0.394369 | 2.531321 | 0.384715 | 0.406036 |
| RT017301 | 0.68784 | 0.802998 | 3.262499 | 0.792833 | 0.591969 | 0.601738 | 3.34551 | 0.610489 | 0.490778 |
| RT017494 | 0.805673 | 1.656632 | 5.021534 | 1.599784 | 0.799972 | 1.262434 | 4.236739 | 1.365838 | 0.838591 |
| RT040223 | 0.871365 | 0.489166 | 7.617426 | 0.451113 | 0.584959 | 0.465808 | 6.626214 | 0.349795 | 0.437507 |
| RT041723 | 1.373056 | 2.375364 | 5.027278 | 0.606121 | 1.423869 | 1.868582 | 3.804584 | 0.333257 | 1.410294 |
| RT041731 | 1.640699 | 0.752594 | 4.311996 | 0.988119 | 1.553059 | 0.541721 | 4.000839 | 0.638473 | 0.488895 |
| RT043133 | 0.526445 | 1.10944 | 13.38216 | 0.586323 | 0.401326 | 0.91524 | 10.42927 | 0.412634 | 0.645961 |
| JI |  |  |  |  |  |  |  |  |  |
| Patient_id | CTV_ALN | CTV_CW | CTV_IMN | CTV_SCN | PTV_ALN | PTV_CW_EVA | PTV_IMN | PTV_SCN | PTVall |
| RT014017 | 0.532087 | 0.387042 | 0.382311 | 0.588255 | 0.63364 | 0.640798 | 0.422617 | 0.64622 | 0.657494 |
| RT014099 | 0.681319 | 0.621895 | 0.353011 | 0.467585 | 0.779837 | 0.753839 | 0.539031 | 0.511773 | 0.734863 |
| RT014263 | 0.096873 | 0.627279 | 0.270069 | 0.573823 | 0.174022 | 0.723393 | 0.399952 | 0.621753 | 0.674587 |
| RT014338 | 0.648148 | 0.541105 | 0.391601 | 0.39471 | 0.692147 | 0.719119 | 0.437774 | 0.433933 | 0.699576 |
| RT014432 | 0.764588 | 0.550543 | 0.228355 | 0.586291 | 0.806275 | 0.6877 | 0.316459 | 0.647592 | 0.699998 |
| RT014482 | 0.761384 | 0.670901 | 0.469975 | 0.557711 | 0.804314 | 0.756626 | 0.453627 | 0.584981 | 0.742214 |
| RT014753 | 0.745678 | 0.584304 | 0.339286 | 0.506975 | 0.794054 | 0.702135 | 0.42966 | 0.492742 | 0.694477 |
| RT014765 | 0.644548 | 0.577197 | 0.363636 | 0.417587 | 0.690964 | 0.715638 | 0.455502 | 0.493258 | 0.70831 |
| RT014819 | 0.767639 | 0.420886 | 0.316402 | 0.559461 | 0.779279 | 0.630788 | 0.453747 | 0.648387 | 0.687966 |
| RT014917 | 0.631165 | 0.56913 | 0.190824 | 0.533008 | 0.732158 | 0.671362 | 0.377267 | 0.616603 | 0.673136 |
| RT014990 | 0.455804 | 0.555944 | 0.26077 | 0.504794 | 0.50393 | 0.691964 | 0.288625 | 0.501744 | 0.669835 |
| RT015074 | 0.551892 | 0.532493 | 0.284335 | 0.414809 | 0.590838 | 0.642226 | 0.390725 | 0.430135 | 0.651147 |
| RT015121 | 0.740137 | 0.573485 | 0.493056 | 0.507171 | 0.791052 | 0.705446 | 0.496686 | 0.584523 | 0.713912 |
| RT015125 | 0.381365 | 0.538006 | 0.350291 | 0.436034 | 0.444389 | 0.747023 | 0.423266 | 0.447215 | 0.694317 |
| RT015385 | 0.649742 | 0.532448 | 0.395991 | 0.50511 | 0.70179 | 0.644691 | 0.454313 | 0.563204 | 0.661382 |
| RT015387 | 0.654158 | 0.532571 | 0.261649 | 0.599446 | 0.68992 | 0.619988 | 0.387741 | 0.677096 | 0.643012 |
| RT015389 | 0.719257 | 0.526787 | 0.537895 | 0.608725 | 0.781814 | 0.709285 | 0.592782 | 0.628892 | 0.726091 |
| RT015442 | 0.682851 | 0.494995 | 0.418304 | 0.506398 | 0.747354 | 0.736214 | 0.488086 | 0.595454 | 0.743104 |
| RT015495 | 0.614396 | 0.52065 | 0.128143 | 0.355502 | 0.653503 | 0.754757 | 0.284687 | 0.397557 | 0.673308 |
| RT015573 | 0.698485 | 0.434674 | 0.299022 | 0.455154 | 0.724524 | 0.649464 | 0.392905 | 0.528318 | 0.668476 |
| RT015833 | 0.591575 | 0.591856 | 0.360451 | 0.604805 | 0.641849 | 0.709721 | 0.456536 | 0.708366 | 0.747423 |
| RT015843 | 0.732562 | 0.504719 | 0.247324 | 0.618032 | 0.773034 | 0.748854 | 0.350707 | 0.667913 | 0.76248 |
| RT016128 | 0.725495 | 0.5762 | 0.449937 | 0.577258 | 0.799098 | 0.709234 | 0.507448 | 0.684756 | 0.737782 |
| RT016140 | 0.664987 | 0.631557 | 0.447438 | 0.558405 | 0.722882 | 0.740416 | 0.44757 | 0.665479 | 0.736215 |
| RT016141 | 0.750971 | 0.649799 | 0.343771 | 0.650958 | 0.786465 | 0.7392 | 0.413911 | 0.69221 | 0.743485 |
| RT016160 | 0.651768 | 0.604385 | 0.373203 | 0.652591 | 0.712215 | 0.735884 | 0.464666 | 0.706476 | 0.750718 |
| RT016223 | 0.708821 | 0.716825 | 0.509215 | 0.626501 | 0.741199 | 0.772016 | 0.519576 | 0.739571 | 0.78357 |
| RT016324 | 0.713503 | 0.567162 | 0.334271 | 0.559714 | 0.736397 | 0.699462 | 0.359612 | 0.637692 | 0.708437 |
| RT016326 | 0.689351 | 0.528691 | 0.393986 | 0.644244 | 0.737663 | 0.715785 | 0.454906 | 0.714483 | 0.742775 |
| RT016334 | 0.676873 | 0.672623 | 0.28892 | 0.6095 | 0.759658 | 0.746615 | 0.417716 | 0.694684 | 0.74686 |
| RT016336 | 0.766204 | 0.668121 | 0.308376 | 0.503671 | 0.813461 | 0.754935 | 0.412277 | 0.658688 | 0.764572 |
| RT016478 | 0.700955 | 0.552098 | 0.355376 | 0.566082 | 0.757252 | 0.638617 | 0.351195 | 0.667505 | 0.666141 |
| RT016541 | 0.55431 | 0.640051 | 0.351099 | 0.665474 | 0.645189 | 0.757028 | 0.413515 | 0.737119 | 0.757167 |
| RT016557 | 0.640817 | 0.661531 | 0.452152 | 0.580422 | 0.664463 | 0.747135 | 0.411877 | 0.636106 | 0.734124 |
| RT016562 | 0.714394 | 0.540968 | 0.433837 | 0.562231 | 0.761635 | 0.65247 | 0.48385 | 0.619967 | 0.689271 |
| RT016705 | 0.634838 | 0.596218 | 0.426307 | 0.655275 | 0.670938 | 0.728311 | 0.473457 | 0.66891 | 0.737322 |
| RT016746 | 0.651695 | 0.661997 | 0.255198 | 0.602375 | 0.707376 | 0.763834 | 0.331394 | 0.703437 | 0.75293 |
| RT016804 | 0.574904 | 0.615215 | 0.366584 | 0.672821 | 0.637963 | 0.711049 | 0.417371 | 0.742657 | 0.735816 |
| RT016814 | 0.604291 | 0.489553 | 0.556145 | 0.509585 | 0.67539 | 0.606355 | 0.547222 | 0.617934 | 0.665364 |
| RT017291 | 0.724299 | 0.545317 | 0.164971 | 0.585924 | 0.814798 | 0.688829 | 0.130042 | 0.669856 | 0.696216 |
| RT017292 | 0.751497 | 0.552602 | 0.4719 | 0.596779 | 0.803079 | 0.663613 | 0.51925 | 0.532103 | 0.683438 |
| RT017299 | 0.61495 | 0.685752 | 0.311313 | 0.674449 | 0.665867 | 0.78345 | 0.437938 | 0.736777 | 0.771422 |
| RT017301 | 0.656794 | 0.659352 | 0.329803 | 0.582122 | 0.704279 | 0.761814 | 0.400516 | 0.674097 | 0.756128 |
| RT017494 | 0.673585 | 0.590214 | 0.339888 | 0.505383 | 0.708838 | 0.713009 | 0.407626 | 0.575658 | 0.704934 |
| RT040223 | 0.589411 | 0.672967 | 0.305339 | 0.708301 | 0.718762 | 0.73796 | 0.386394 | 0.785283 | 0.76315 |
| RT041723 | 0.623181 | 0.393494 | 0.41166 | 0.623765 | 0.683148 | 0.598972 | 0.526812 | 0.78018 | 0.676191 |
| RT041731 | 0.597265 | 0.602634 | 0.336418 | 0.548753 | 0.653405 | 0.731161 | 0.461502 | 0.701945 | 0.771822 |
| RT043133 | 0.708673 | 0.628083 | 0.22612 | 0.620446 | 0.778394 | 0.723253 | 0.39603 | 0.733899 | 0.766025 |
