# Supplementary material for: Geometric and dosimetric evaluation of deep learning based auto‐segmentation for clinical target volume on breast cancer
Source: J Appl Clin Med Phys. 2023 Mar 15;24(7):e13951. doi: 10.1002/acm2.13951 (PMC10338811; doi:10.1002/acm2.13951)
Supplement: Supplementary file 4 — Supporting Information [file ACM2-24-e13951-s004.docx]

| **Unit：（cGy)** | **RT014017** | | **RT014099** | | **RT014263** | | **RT014338** | | **RT014432** | | **RT014482** | |
| --- | --- | --- | --- | --- | --- | --- | --- | --- | --- | --- | --- | --- |
|  | **Manual** | **Auto** | **Manual** | **Auto** | **Manual** | **Auto** | **Manual** | **Auto** | **Manual** | **Auto** | **Manual** | **Auto** |
| **BREAST_CON V5** | 20.54 | 26.84 | 4.02 | 6.54 | 13 | 15.84 | 8.21 | 9.16 | 5.47 | 5.4 | 6.67 | 9.06 |
| **Esophagus Dmean** | 539.08 | 581.07 | 410.23 | 546.89 | 257.06 | 347.34 | 375.78 | 625.03 | 574.9 | 1029.97 | 1199.24 | 1334.01 |
| **Heart Dmean** | 290.14 | 290.73 | 596.27 | 672.61 | 615.3 | 689.44 | 612.42 | 590.42 | 220.41 | 234.53 | 525.93 | 593.11 |
| **Humeralhead Dmean** | 2491.16 | 2465.11 | 2209.83 | 2053.97 | 1904 | 1857.1 | 2226.84 | 2257.52 | 2231.31 | 2247.49 | 2241.98 | 2312.8 |
| **Lung V5** | 58.68 | 58.99 | 57.02 | 56.89 | 54.92 | 55.11 | 54.24 | 55.23 | 60.33 | 59.78 | 55.01 | 54.84 |
| **Lung V20** | 33.49 | 32.75 | 28.26 | 28.86 | 26.03 | 27.36 | 26.67 | 27.12 | 28.36 | 27.63 | 26.42 | 27.24 |
| **SpinalCord Dmax** | 672.04 | 696.59 | 1686.94 | 2172.08 | 1313.76 | 2291.45 | 688.81 | 1157.52 | 913.85 | 1032.33 | 833.34 | 973.07 |
| **Thyroid Dmean** | 2508.31 | 2497.86 | 2278.19 | 2433.12 | 2361.38 | 2430.44 | 2189.14 | 2485.33 | 2470.97 | 2554.27 | 2262.34 | 2408.69 |
| **Dmax** | 5938.7 | 5779.33 | 5729.61 | 5793.2 | 5730.7 | 5880.78 | 5635.76 | 5864.3 | 5806.42 | 5710.2 | 5610.94 | 5659.85 |
| **Dmin** | 1771.4 | 1017.95 | 3712.2 | 1663.79 | 3335.68 | 629.04 | 3458.65 | 1318.9 | 3087.41 | 1711.64 | 4121.69 | 2875.34 |
| **Dmean** | 5259.62 | 5078.44 | 5220.85 | 5203.58 | 5197.65 | 5087.03 | 5198 | 5152.4 | 5219.75 | 5112.64 | 5204.48 | 5157.73 |
| **D5** | 5506.99 | 5432.7 | 5430.94 | 5481.83 | 5394.6 | 5466.82 | 5395.11 | 5428.95 | 5439.77 | 5440.49 | 5394.64 | 5374.78 |
| **D95** | 5000 | 4217.31 | 5000 | 4790.2 | 5000 | 3968.59 | 5000 | 4616.56 | 5000 | 4466.56 | 5000 | 4938.65 |
| **Vptv of 95%** | 673.1 | 606.97 | 515.75 | 494.73 | 612.33 | 560.98 | 513.26 | 482.24 | 743.88 | 680.53 | 406.5 | 404.72 |
| **Vbody of 95%** | 946.72 | 880.43 | 700.68 | 746.65 | 778.19 | 878.61 | 688.08 | 732.45 | 956.1 | 911.96 | 553.93 | 649.66 |
| **Vptv** | 683.946 | 683.946 | 518.212 | 518.212 | 614.656 | 614.656 | 514.963 | 514.963 | 748.198 | 748.198 | 407.438 | 407.438 |
| **CI** | 0.699706 | 0.61181174 | 0.7325736 | 0.632575 | 0.783887 | 0.582729 | 0.743464 | 0.616556 | 0.773546 | 0.678738 | 0.732158 | 0.618816 |
| **HI** | 1.101398 | 1.28819081 | 1.086188 | 1.144384 | 1.07892 | 1.377522 | 1.079022 | 1.175973 | 1.087954 | 1.218049 | 1.078928 | 1.08831 |
|  | **RT014753** | | **RT014765** | | **RT014819** | | **RT014917** | | **RT014990** | | **RT015074** | |
|  | **Manual** | **Auto** | **Manual** | **Auto** | **Manual** | **Auto** | **Manual** | **Auto** | **Manual** | **Auto** | **Manual** | **Auto** |
| **BREAST_CON V5** | 3.26 | 2.94 | 12.2 | 9.52 | 9.57 | 5.6 | 11.49 | 11.97 | 10.04 | 9.76 | 13.09 | 10.51 |
| **Esophagus Dmean** | 452.18 | 562.51 | 813.69 | 961.6 | 772.31 | 893.93 | 726.27 | 922.44 | 370 | 615.58 | 381.03 | 525.78 |
| **Heart Dmean** | 299.69 | 302.37 | 602.71 | 558.95 | 304.03 | 289.65 | 259.58 | 257.86 | 264.81 | 272.95 | 306.71 | 316.55 |
| **Humeralhead Dmean** | 2184.57 | 2248.02 | 2067.43 | 1988.28 | 2333.22 | 2326.31 | 1978.1 | 2248.25 | 2321.39 | 2269.38 | 887.93 | 1314.75 |
| **Lung V5** | 59.94 | 59.29 | 57.75 | 57.19 | 59.24 | 58.97 | 59.19 | 60.42 | 60.95 | 59.19 | 62.8 | 63.74 |
| **Lung V20** | 32.08 | 31.52 | 28.8 | 29.41 | 31.63 | 31.11 | 27.92 | 28.04 | 27.61 | 28.23 | 32.09 | 32.63 |
| **SpinalCord Dmax** | 1443.98 | 1638.52 | 1114.38 | 1259.48 | 1267.44 | 1847.19 | 1096.62 | 829.44 | 683.03 | 1052.44 | 1227.73 | 1818.06 |
| **Thyroid Dmean** | 1869.43 | 2265.92 | 2014.33 | 2358.85 | 2113.21 | 1363.26 | 2277.48 | 2328.5 | 686.69 | 2329.79 | 2250.84 | 2291.99 |
| **Dmax** | 5767.27 | 5763.8 | 5795.44 | 5989.32 | 5707.96 | 5812.53 | 5678.17 | 5800.81 | 5735.29 | 5808.2 | 5848.8 | 6044.92 |
| **Dmin** | 3502.03 | 924.49 | 3509.33 | 725.24 | 3577.38 | 543.51 | 3432.62 | 1475.36 | 3348.82 | 1134.95 | 3470.94 | 564.36 |
| **Dmean** | 5249.95 | 5087.97 | 5242.63 | 5156.31 | 5235.55 | 5026.02 | 5221.69 | 5040.38 | 5226.22 | 5103.99 | 5264.46 | 5002.26 |
| **D5** | 5484.92 | 5478.82 | 5472.17 | 5512.35 | 5445.52 | 5442.56 | 5433.87 | 5441.63 | 5447.2 | 5461.1 | 5526.96 | 5517.39 |
| **D95** | 5000 | 3876.87 | 5000 | 4487.58 | 5000 | 3662.39 | 5000 | 3748.57 | 5000 | 4263.66 | 5000 | 3485.01 |
| **Vptv of 95%** | 617.15 | 561.05 | 438.76 | 410.04 | 459.05 | 400.52 | 606.7 | 529.4 | 592.83 | 538.76 | 679.32 | 578.19 |
| **Vbody of 95%** | 833.75 | 817.78 | 618.57 | 635.7 | 651.71 | 592.17 | 796.89 | 731.77 | 774.03 | 792.03 | 966.46 | 859.33 |
| **Vptv** | 620.309 | 620.309 | 441.298 | 441.298 | 461.531 | 461.531 | 609.533 | 609.533 | 595.656 | 595.656 | 685.514 | 685.514 |
| **CI** | 0.73644 | 0.620524 | 0.705234 | 0.599333 | 0.700591 | 0.58695 | 0.757796 | 0.628342 | 0.762267 | 0.615253 | 0.696544 | 0.567499 |
| **HI** | 1.096984 | 1.413207 | 1.094434 | 1.228357 | 1.089104 | 1.486068 | 1.086774 | 1.451655 | 1.08944 | 1.280848 | 1.105392 | 1.583178 |
|  | **RT015121** | | **RT015125** | | **RT015385** | | **RT015387** | | **RT015389** | | **RT015442** | |
|  | **Manual** | **Auto** | **Manual** | **Auto** | **Manual** | **Auto** | **Manual** | **Auto** | **Manual** | **Auto** | **Manual** | **Auto** |
| **BREAST_CON V5** | 5.63 | 8.29 | 13.51 | 9.88 | 5.63 | 8.36 | 2.81 | 3.12 | 17.73 | 17.74 | 38.74 | 39.08 |
| **Esophagus Dmean** | 539.85 | 650.33 | 214.38 | 248.94 | 912.08 | 2061.89 | 237.59 | 316.23 | 431.89 | 807.35 | 688.29 | 910.24 |
| **Heart Dmean** | 530.41 | 523.76 | 263.92 | 258.3 | 585.27 | 603.04 | 297.89 | 288.21 | 202.01 | 184.06 | 262.29 | 264.85 |
| **Humeralhead Dmean** | 2396.41 | 2428.53 | 2399.5 | 2548.74 | 2061.81 | 2215.21 | 2360.52 | 2380.95 | 2390.25 | 2429.32 | 2248.75 | 2275.44 |
| **Lung V5** | 54.68 | 54.87 | 59.32 | 59.46 | 52.65 | 53.25 | 60.72 | 60.3 | 60.27 | 61.9 | 61.2 | 61.56 |
| **Lung V20** | 29.36 | 29.69 | 28.69 | 28.41 | 22.64 | 24.28 | 29.76 | 30.17 | 29.9 | 30.49 | 28.37 | 28.31 |
| **SpinalCord Dmax** | 970.31 | 1011.16 | 1052.81 | 1098.64 | 662.07 | 1458.7 | 929.6 | 798.96 | 547.11 | 806.27 | 751.37 | 1017.91 |
| **Thyroid Dmean** | 2308.91 | 2439.21 | 609.16 | 2413.9 | 2107.47 | 2278.21 | 2292.36 | 2305.29 | 2223.06 | 2440.44 | 2221.48 | 2298.35 |
| **Dmax** | 5645.09 | 5788.29 | 5743.81 | 5843.8 | 5665.07 | 5661.34 | 5773.31 | 5691.56 | 5576.08 | 5738.38 | 5677.26 | 5691.89 |
| **Dmin** | 3769.57 | 1673.84 | 3334.82 | 837.46 | 3596.86 | 1746.32 | 3549.49 | 968.56 | 3996.96 | 1738.41 | 3322.92 | 752.84 |
| **Dmean** | 5198.71 | 5138.53 | 5241.09 | 5179.59 | 5198.91 | 5174.34 | 5207.97 | 5030.91 | 5212.74 | 5145.19 | 5220.18 | 5131.01 |
| **D5** | 5406.4 | 5449.14 | 5461.18 | 5481.42 | 5384.4 | 5410.13 | 5416.07 | 5426.83 | 5406.08 | 5444.42 | 5428.75 | 5422.61 |
| **D95** | 5000 | 4627.09 | 5000 | 4679.05 | 5000 | 4811.57 | 5000 | 3746.8 | 5000 | 4581.11 | 5000 | 4513.89 |
| **Vptv of 95%** | 728.87 | 681.04 | 557.07 | 527.49 | 530.62 | 510.69 | 705.79 | 612.87 | 515.5 | 482.55 | 649.31 | 606.85 |
| **Vbody of 95%** | 925.7 | 975.81 | 732.37 | 797.58 | 686.31 | 830.23 | 934.9 | 934.5 | 675.1 | 683.45 | 837.98 | 831.77 |
| **Vptv** | 731.696 | 731.696 | 560.089 | 560.089 | 532.312 | 532.312 | 708.23 | 708.23 | 517.007 | 517.007 | 652.684 | 652.684 |
| **CI** | 0.784331 | 0.649605 | 0.75654 | 0.62287 | 0.770692 | 0.590133 | 0.752335 | 0.567523 | 0.761365 | 0.658994 | 0.770846 | 0.678354 |
| **HI** | 1.08128 | 1.17766 | 1.092236 | 1.171481 | 1.07688 | 1.1244 | 1.083214 | 1.448391 | 1.081216 | 1.18845 | 1.08575 | 1.201316 |
|  | **RT015495** | | **RT015573** | | **RT015833** | | **RT015843** | | **RT016128** | | **RT016140** | |
|  | **Manual** | **Auto** | **Manual** | **Auto** | **Manual** | **Auto** | **Manual** | **Auto** | **Manual** | **Auto** | **Manual** | **Auto** |
| **BREAST_CON V5** | 11.21 | 12.82 | 2.7 | 2.09 | 24.31 | 27.36 | 0.07 | 0 | 0 | 0.16 | 11.6 | 16.32 |
| **Esophagus Dmean** | 1697.2 | 1574.17 | 380.74 | 507.83 | 1089.17 | 1207.95 | 296.65 | 348.32 | 722.13 | 1039.25 | 917.08 | 1228.86 |
| **Heart Dmean** | 775.77 | 855.19 | 257.66 | 255.3 | 663.49 | 708.5 | 258.16 | 255.14 | 735.8 | 769.59 | 709.84 | 871.16 |
| **Humeralhead Dmean** | 2231.93 | 2118.74 | 2216.13 | 2268.43 | 2342.5 | 2510.99 | 2120.01 | 2252.13 | 2251.46 | 2254.92 | 2184.64 | 2274.12 |
| **Lung V5** | 53.4 | 54.32 | 58.34 | 58.92 | 54.25 | 54.35 | 57.37 | 56.79 | 53.59 | 54.1 | 55.26 | 56.11 |
| **Lung V20** | 24.55 | 26.83 | 30.57 | 30.44 | 27.2 | 27.6 | 28.88 | 28.12 | 27.01 | 27.5 | 28.13 | 29.23 |
| **SpinalCord Dmax** | 2446.1 | 1397.32 | 884.3 | 910.45 | 784.28 | 878.8 | 1038.28 | 1072.16 | 850.01 | 1523.98 | 989.95 | 871.46 |
| **Thyroid Dmean** | 2271.58 | 2367.38 | 2245.44 | 2383.18 | 2231.01 | 2289.41 | 2187 | 2281.06 | 2204.36 | 2369.06 | 283.02 | 2397.43 |
| **Dmax** | 5714.51 | 5931 | 5814.11 | 5826.27 | 5699.27 | 5753.79 | 5736.17 | 5787.14 | 5697.38 | 5793.01 | 5689.43 | 5911.24 |
| **Dmin** | 2888.92 | 290.08 | 3198.52 | 1555.99 | 2794.46 | 1370.01 | 3869.7 | 1352.71 | 2902.53 | 2210.29 | 3026.44 | 885.48 |
| **Dmean** | 5216.07 | 4923.04 | 5215.73 | 4993.98 | 5201.94 | 5144.88 | 5225.58 | 5203.1 | 5231.84 | 5195.81 | 5229.98 | 5262.6 |
| **D5** | 5421.91 | 5462.14 | 5440.29 | 5434.55 | 5400.51 | 5431.44 | 5428.15 | 5450.49 | 5446.43 | 5500.86 | 5435.44 | 5526.69 |
| **D95** | 5000 | 2896.56 | 5000 | 3734.81 | 5000 | 4521.67 | 5000 | 4883.11 | 5000 | 4683.39 | 5000 | 4943.46 |
| **Vptv of 95%** | 555.69 | 480.67 | 819.4 | 690.33 | 668.46 | 622.54 | 420.39 | 406.77 | 633.72 | 599.88 | 412.63 | 403.78 |
| **Vbody of 95%** | 750.87 | 727.02 | 1031.51 | 863.73 | 837.7 | 842.83 | 572.08 | 602.35 | 827.24 | 822.89 | 599.7 | 676.8 |
| **Vptv** | 558.866 | 558.866 | 824.605 | 824.605 | 670.934 | 670.934 | 421.634 | 421.634 | 637.501 | 637.501 | 414.722 | 414.722 |
| **CI** | 0.735856 | 0.568643 | 0.789355 | 0.669098 | 0.795028 | 0.685354 | 0.732677 | 0.651498 | 0.761522 | 0.685972 | 0.68459 | 0.580861 |
| **HI** | 1.084382 | 1.885733 | 1.088058 | 1.455107 | 1.080102 | 1.201202 | 1.08563 | 1.116192 | 1.089286 | 1.174547 | 1.087088 | 1.11798 |
|  | **RT016141** | | **RT016160** | | **RT016223** | | **RT016324** | | **RT016326** | | **RT016334** | |
|  | **Manual** | **Auto** | **Manual** | **Auto** | **Manual** | **Auto** | **Manual** | **Auto** | **Manual** | **Auto** | **Manual** | **Auto** |
| **BREAST_CON V5** | 8.28 | 12.01 | 4.29 | 5.12 | 1.99 | 3.01 | 1.47 | 0 | 0 | 0 | 0 | 0.02 |
| **Esophagus Dmean** | 2206.47 | 2225.14 | 1150.32 | 1589.23 | 2190.58 | 2247.23 | 1420.08 | 2054.57 | 379.13 | 603.02 | 1053.58 | 961.33 |
| **Heart Dmean** | 576.76 | 623.81 | 608.73 | 625.95 | 593.13 | 608.22 | 788.23 | 776.44 | 176.53 | 185.58 | 697.55 | 746.08 |
| **Humeralhead Dmean** | 2207.66 | 2198.42 | 2146.05 | 2252.1 | 2452.63 | 2428.17 | 2280.16 | 2312.99 | 1357.24 | 1398.42 | 2094.12 | 1962.05 |
| **Lung V5** | 54.02 | 55.11 | 56.95 | 58.83 | 55.42 | 55.55 | 58.93 | 60.87 | 58.67 | 57.97 | 55.2 | 56.24 |
| **Lung V20** | 27.72 | 28.14 | 28.04 | 28.55 | 27.58 | 28.02 | 31.12 | 32.59 | 27.05 | 27.11 | 27.91 | 28.75 |
| **SpinalCord Dmax** | 707.43 | 850.13 | 1244.59 | 1540.22 | 1211.13 | 1740.85 | 621.96 | 820.73 | 1635.4 | 1375.33 | 1006 | 1136.43 |
| **Thyroid Dmean** | 2046.76 | 2227.75 | 2254.64 | 2382.51 | 2465.35 | 2561.39 | 2824.56 | 3043.28 | 2257.01 | 2285.72 | 2420.2 | 2537.44 |
| **Dmax** | 5534.32 | 5668.26 | 5719.58 | 5776.18 | 5680.26 | 5729.92 | 5527.47 | 5563.42 | 5665.67 | 5754.1 | 5710.73 | 5854.75 |
| **Dmin** | 4166.68 | 953.58 | 3692.43 | 1053.75 | 3852.52 | 1435.33 | 3059.56 | 986.36 | 1736.71 | 1605.09 | 3168.15 | 696.48 |
| **Dmean** | 5181.4 | 5190.15 | 5219.09 | 5181.78 | 5214.04 | 5209.03 | 5144.17 | 5105.89 | 5206 | 5142.57 | 5231.78 | 5248.76 |
| **D5** | 5351.04 | 5415.54 | 5430.99 | 5479.37 | 5406.7 | 5444.28 | 5271.32 | 5318.83 | 5402.97 | 5432.19 | 5449.35 | 5526.9 |
| **D95** | 5000 | 4912.59 | 5000 | 4715.97 | 5000 | 4918.68 | 5000 | 4648.51 | 5000 | 4618.11 | 5000 | 4929.62 |
| **Vptv of 95%** | 445.73 | 432.69 | 743.61 | 704.55 | 517.85 | 502.99 | 648.55 | 612.11 | 556.21 | 523.05 | 424.64 | 413.82 |
| **Vbody of 95%** | 584.45 | 678.3 | 956.33 | 924.81 | 689.85 | 756.58 | 919.58 | 902.65 | 743.46 | 771.79 | 605.4 | 670.73 |
| **Vptv** | 446.209 | 446.209 | 746.387 | 746.387 | 519.755 | 519.755 | 652.805 | 652.805 | 557.893 | 557.893 | 426.939 | 426.939 |
| **CI** | 0.76183 | 0.618577 | 0.774673 | 0.719129 | 0.747919 | 0.643376 | 0.700671 | 0.635852 | 0.74588 | 0.635384 | 0.697644 | 0.598011 |
| **HI** | 1.070208 | 1.10238 | 1.086198 | 1.161875 | 1.08134 | 1.106858 | 1.054264 | 1.144201 | 1.080594 | 1.17628 | 1.08987 | 1.121161 |
|  | **RT016336** | | **RT016478** | | **RT016541** | | **RT016557** | | **RT016562** | | **RT016705** | |
|  | **Manual** | **Auto** | **Manual** | **Auto** | **Manual** | **Auto** | **Manual** | **Auto** | **Manual** | **Auto** | **Manual** | **Auto** |
| **BREAST_CON V5** | 9.14 | 10.14 | 2.09 | 5.45 | 12.81 | 13.81 | 5.76 | 9.38 | 1.7 | 1.71 | 2.4 | 1.06 |
| **Esophagus Dmean** | 1964.56 | 1856.66 | 697.04 | 803.28 | 428.89 | 563.15 | 1358.34 | 1623.12 | 275.37 | 715.66 | 359.5 | 561.08 |
| **Heart Dmean** | 568.26 | 638.82 | 287.05 | 303.48 | 267.95 | 293.37 | 611.34 | 652.38 | 137.54 | 139.03 | 246.51 | 244.05 |
| **Humeralhead Dmean** | 2418.8 | 2438.84 | 1718.14 | 1574.93 | 2137.95 | 2248.79 | 1892.93 | 2069.49 | 2017.45 | 2080.1 | 2258.63 | 2246.16 |
| **Lung V5** | 54.23 | 55.08 | 60.21 | 62.53 | 61.29 | 62.45 | 56.42 | 57.59 | 61.22 | 61.65 | 57.4 | 56.3 |
| **Lung V20** | 26.95 | 27.28 | 30.22 | 31.3 | 31.72 | 32.34 | 27.27 | 27.84 | 33.05 | 33.17 | 27.29 | 27.32 |
| **SpinalCord Dmax** | 806.22 | 697.19 | 881.11 | 1757.91 | 1232.04 | 1088.13 | 735.47 | 1079.19 | 243.91 | 384.08 | 1324.51 | 2080.2 |
| **Thyroid Dmean** | 2374.18 | 2491.17 | 2540.53 | 2581.63 | 2521 | 2623.66 | 2330.89 | 2371.68 | 2794.03 | 3437.8 | 2279.13 | 2411.97 |
| **Dmax** | 5652.12 | 5772.69 | 5797 | 6058.71 | 5769.15 | 5907.04 | 5616.34 | 5777.4 | 5848.15 | 5956.06 | 5562.81 | 5643.06 |
| **Dmin** | 3572.18 | 770.51 | 1678.12 | 1542.02 | 3877.56 | 1739.49 | 2942.5 | 900.07 | 1709.34 | 583.83 | 4002.59 | 1995.89 |
| **Dmean** | 5201.59 | 5177.53 | 5233.65 | 4977.38 | 5236.6 | 5125.43 | 5205.72 | 5194.11 | 5262.54 | 5124.27 | 5198.83 | 5128.25 |
| **D5** | 5386.87 | 5456.11 | 5470.83 | 5464.84 | 5466.64 | 5470.46 | 5400.84 | 5461.34 | 5513.13 | 5568.77 | 5385.28 | 5413.07 |
| **D95** | 5000 | 4749.52 | 5000 | 3539.86 | 5000 | 4317.28 | 5000 | 4817.33 | 5000 | 3850.23 | 5000 | 4610.27 |
| **Vptv of 95%** | 615.35 | 586.29 | 980.35 | 805.83 | 804.98 | 730.29 | 474.88 | 457.34 | 557.39 | 495.68 | 684.08 | 635.79 |
| **Vbody of 95%** | 782.1 | 808.11 | 1193.69 | 1069.47 | 1051.05 | 945.27 | 632.36 | 695.79 | 819.78 | 761.14 | 864.22 | 845.49 |
| **Vptv** | 617.179 | 617.179 | 989.255 | 989.255 | 808.226 | 808.226 | 476.589 | 476.589 | 562.283 | 562.283 | 685.708 | 685.708 |
| **CI** | 0.78446 | 0.689197 | 0.813884 | 0.613776 | 0.762806 | 0.698075 | 0.748272 | 0.630748 | 0.67401 | 0.574094 | 0.789678 | 0.697236 |
| **HI** | 1.077374 | 1.148771 | 1.094166 | 1.543801 | 1.093328 | 1.267108 | 1.080168 | 1.133686 | 1.102626 | 1.446347 | 1.077056 | 1.174133 |
|  | **RT016746** | | **RT016804** | | **RT016814** | | **RT017291** | | **RT017292** | | **RT017299** | |
|  | **Manual** | **Auto** | **Manual** | **Auto** | **Manual** | **Auto** | **Manual** | **Auto** | **Manual** | **Auto** | **Manual** | **Auto** |
| **BREAST_CON V5** | 9.27 | 8.99 | 5.99 | 7.34 | 4.49 | 5.1 | 0.04 | 0 | 0 | 0.01 | 15.13 | 20.96 |
| **Esophagus Dmean** | 457.59 | 608.12 | 1398.02 | 1412.86 | 339.36 | 484.46 | 495.2 | 565.09 | 317.51 | 614.61 | 1168.13 | 1656.21 |
| **Heart Dmean** | 258.79 | 257.24 | 734.18 | 788.27 | 665.33 | 666.7 | 292.56 | 268.92 | 263.54 | 267.05 | 771.98 | 961.84 |
| **Humeralhead Dmean** | 2007 | 2214 | 2093.08 | 2126.41 | 2229.18 | 2236.75 | 1619.2 | 1554.08 | 2234.73 | 2279.54 | 2124.68 | 2363.97 |
| **Lung V5** | 57.24 | 57.13 | 59.55 | 60.7 | 55.78 | 56.32 | 63.67 | 60.99 | 59.11 | 58.74 | 55.93 | 59.05 |
| **Lung V20** | 31.16 | 30.66 | 32 | 32.4 | 28.72 | 28.26 | 33.98 | 33.12 | 27.88 | 28.49 | 29.81 | 31.45 |
| **SpinalCord Dmax** | 1332.39 | 1505.71 | 1235.98 | 1768.98 | 794.94 | 430.56 | 1062.52 | 1113.11 | 690.56 | 1086.83 | 598.58 | 524.9 |
| **Thyroid Dmean** | 2353.23 | 2365.11 | 2435.39 | 2461.25 | 2228.52 | 2490.44 | 2373.83 | 2445.48 | 1901.2 | 2497.03 | 2388.34 | 2804.3 |
| **Dmax** | 5708.81 | 5683.06 | 5675.64 | 5801.7 | 5647.43 | 5795.1 | 5857.75 | 6002.95 | 5703.99 | 5877.67 | 5708.49 | 5644.52 |
| **Dmin** | 3693.72 | 1420.35 | 3502.36 | 1904.35 | 3528.46 | 1269.54 | 3565.5 | 546.64 | 3247.68 | 1479.13 | 1740.07 | 1730.43 |
| **Dmean** | 5210.85 | 5189.97 | 5216.91 | 5127.47 | 5222.37 | 5056.89 | 5251.58 | 5108.09 | 5233.23 | 5147.43 | 5222.98 | 5162.77 |
| **D5** | 5415.86 | 5422.26 | 5417.24 | 5447.24 | 5441.69 | 5475.66 | 5494.87 | 5489.15 | 5451.88 | 5514.76 | 5435.29 | 5344.39 |
| **D95** | 5000 | 4842.8 | 5000 | 4321.74 | 5000 | 3778.82 | 5000 | 4222.22 | 5000 | 4425.87 | 5000 | 4953.28 |
| **Vptv of 95%** | 372.28 | 359.16 | 533.54 | 493.12 | 625.42 | 548.9 | 519.27 | 475.3 | 535.26 | 495.3 | 502.95 | 495.43 |
| **Vbody of 95%** | 529.62 | 550.61 | 720.47 | 729.46 | 843.26 | 766.36 | 752.38 | 739.86 | 738.39 | 781.62 | 685.65 | 812.86 |
| **Vptv** | 373.758 | 373.758 | 535.487 | 729.46 | 628.221 | 628.221 | 524.01 | 524.01 | 537.359 | 537.359 | 505.468 | 505.468 |
| **CI** | 0.700139 | 0.626818 | 0.737852 | 0.456985 | 0.738362 | 0.625808 | 0.683927 | 0.582702 | 0.72207 | 0.584086 | 0.729883 | 0.597386 |
| **HI** | 1.083172 | 1.119654 | 1.083448 | 1.260428 | 1.088338 | 1.44904 | 1.098974 | 1.300063 | 1.090376 | 1.246028 | 1.087058 | 1.07896 |
|  | **RT017301** | | **RT017494** | | **RT040223** | | **RT041723** | | **RT041731** | | **RT043133** | |
|  | **Manual** | **Auto** | **Manual** | **Auto** | **Manual** | **Auto** | **Manual** | **Auto** | **Manual** | **Auto** | **Manual** | **Auto** |
| **BREAST_CON V5** | 3.28 | 4.92 | 4.8 | 2.56 | 12.77 | 13.02 | 14.28 | 14.61 | 1.29 | 1.12 | 25.98 | 25.33 |
| **Esophagus Dmean** | 418.31 | 568.54 | 513.63 | 828.41 | 596.28 | 660.02 | 433.14 | 442.35 | 65.27 | 63.49 | 576.45 | 678.04 |
| **Heart Dmean** | 216.69 | 239.25 | 255.97 | 257.03 | 588.95 | 607.22 | 156.33 | 157.4 | 164.28 | 156.04 | 523.21 | 530.42 |
| **Humeralhead Dmean** | 2227.66 | 2264.77 | 2211.58 | 2256.93 | 1284.94 | 1441.42 | 2084.03 | 2092.17 | 2076.21 | 2228.04 | 1228.48 | 1335.52 |
| **Lung V5** | 58.69 | 59.38 | 55.84 | 58.27 | 55.4 | 55.74 | 58.29 | 59.7 | 55.77 | 54.36 | 49.51 | 50.46 |
| **Lung V20** | 30.28 | 30.81 | 27.28 | 28.05 | 26.87 | 27.46 | 30.57 | 31.39 | 28.66 | 27.88 | 23.89 | 24.4 |
| **SpinalCord Dmax** | 1252.43 | 1422.16 | 692.26 | 1456.53 | 673.53 | 791.09 | 1025.11 | 961.19 | 656.04 | 768.76 | 352.56 | 655.02 |
| **Thyroid Dmean** | 2364.74 | 2497.1 | 2245.12 | 2460.64 | 2062.16 | 2072.86 | 2266.51 | 2262.42 | 2108.62 | 2043.92 | 2048.79 | 2099.88 |
| **Dmax** | 5808.63 | 5905.72 | 5648.29 | 5793.16 | 5709.11 | 5698.46 | 5698.49 | 5772.38 | 5781.99 | 5693.77 | 5637.69 | 5679.73 |
| **Dmin** | 3709.96 | 1189.24 | 4002.4 | 1822.77 | 2699.81 | 1418.78 | 1730.26 | 858.4 | 2910.05 | 443.25 | 3153.9 | 1667.76 |
| **Dmean** | 5219.41 | 5199.6 | 5203.56 | 5152.03 | 5235.02 | 5236.04 | 5226.22 | 5216.31 | 5241.51 | 5142.28 | 5217.56 | 5195.9 |
| **D5** | 5424.42 | 5456.86 | 5392.99 | 5434.99 | 5451.9 | 5470.42 | 5431.73 | 5487.2 | 5458.42 | 5423.62 | 5417.06 | 5438.7 |
| **D95** | 5000 | 4799.85 | 5000 | 4682.36 | 5000 | 4969.5 | 5000 | 4945.65 | 5000 | 4834.48 | 5000 | 4890.82 |
| **Vptv of 95%** | 536.73 | 514.77 | 477.89 | 451.8 | 506.17 | 494.73 | 493.63 | 480.49 | 531.8 | 512.23 | 651.81 | 628.44 |
| **Vbody of 95%** | 714.76 | 771.33 | 635.33 | 694.13 | 669.64 | 772.41 | 678.7 | 864.95 | 815.13 | 829.27 | 811.1 | 926.62 |
| **Vptv** | 538.527 | 538.527 | 479.02 | 479.02 | 506.169 | 506.169 | 495.434 | 495.434 | 535.292 | 535.292 | 653.111 | 653.111 |
| **CI** | 0.748418 | 0.637938 | 0.750417 | 0.613901 | 0.755885 | 0.626027 | 0.724669 | 0.538756 | 0.648155 | 0.591076 | 0.802012 | 0.652588 |
| **HI** | 1.084884 | 1.136881 | 1.078598 | 1.160737 | 1.09038 | 1.100799 | 1.086346 | 1.1095 | 1.091684 | 1.121862 | 1.083412 | 1.112022 |
